# Inhibition of HECT E3 ligases as potential therapy for COVID-19

**DOI:** 10.1038/s41419-021-03513-1

**Published:** 2021-03-24

**Authors:** Giuseppe Novelli, Jing Liu, Michela Biancolella, Tonino Alonzi, Antonio Novelli, J. J. Patten, Dario Cocciadiferro, Emanuele Agolini, Vito Luigi Colona, Barbara Rizzacasa, Rosalinda Giannini, Benedetta Bigio, Delia Goletti, Maria Rosaria Capobianchi, Sandro Grelli, Justin Mann, Trevor D. McKee, Ke Cheng, Fatima Amanat, Florian Krammer, Andrea Guarracino, Gerardo Pepe, Carlo Tomino, Yacine Tandjaoui-Lambiotte, Yurdagul Uzunhan, Sarah Tubiana, Jade Ghosn, COVID Human Genetic Effort, French COVID Cohort Study Group, CoV-Contact Cohort, Luigi D. Notarangelo, Helen C. Su, Laurent Abel, Aurélie Cobat, Gai Elhanan, Joseph J. Grzymski, Andrea Latini, Sachdev S. Sidhu, Suresh Jain, Robert A. Davey, Jean-Laurent Casanova, Wenyi Wei, Pier Paolo Pandolfi

**Affiliations:** 1 Department of Biomedicine and Prevention, Tor Vergata University of Rome, 00133 Rome, Italy; 2 IRCCS Neuromed, Pozzilli, (IS) Italy; 3 Department of Pharmacology, School of Medicine, University of Nevada, Reno, NV 89557 USA; 4 Department of Pathology, Beth Israel Deaconess Cancer Center, Harvard Medical School, Boston, MA 02215 USA; 5 Department of Biology, Tor Vergata University, 00133 Rome, Italy; 6 Translational Research Unit, Department of Epidemiology and Preclinical Research, National Institute for Infectious Diseases Lazzaro Spallanzani – IRCCS, 00149 Rome, Italy; 7 Laboratory of Medical Genetics, IRCCS Bambino Gesù Children’s Hospital, 00165 Rome, Italy; 8 National Emerging Infectious Diseases Laboratories, Boston University, Boston, MA USA; 9 St. Giles Laboratory of Human Genetics of Infectious Diseases, Rockefeller Branch, Rockefeller University, New York, NY 10065 USA; 10 Laboratory of Virology, Department of Epidemiology and Preclinical Research, National Institute for Infectious Diseases Lazzaro Spallanzani – IRCCS, 00149 Rome, Italy; 11 Department of Experimental Medicine, Tor Vergata University of Rome, 00133 Rome, Italy; 12 HistoWiz Inc, Brooklyn, NY 11226 USA; 13 Department of Microbiology, Icahn school of Medicine at Mount Sinai, New York, NY 10029 USA; 14 San Raffaele University of Rome, 00166 Rome, Italy; 15 Intensive Care Unit, Avicenne Hospital, APHP, Bobigny, France; 16 INSERM U1272 Hypoxia & Lung, Bobigny, France; 17 Pneumology Department, Reference Center for Rare Pulmonary Diseases, Hôpital Avicenne, APHP, Bobigny; INSERM UMR1272, Université Paris 13, Bobigny, France; 18 Hôpital Bichat Claude Bernard, APHP, Paris, France; 19 Centre d’investigation Clinique, Inserm CIC, 1425 Paris, France; 20 Infection, Antimicrobials, Modelling, Evolution (IAME), INSERM, UMRS1137, University of Paris, Paris, France; 21 AP-HP, Bichat Claude Bernard Hospital, Infectious and Tropical Disease Department, Paris, France; 22 Laboratory of Clinical Immunology, NIAID, NIH, Bethesda, MD USA; 23 Laboratory of Human Genetics of Infectious Diseases, Necker Branch, INSERM, Necker Hospital for Sick Children, Paris, France; 24 University of Paris, Imagine Institute, Paris, France; 25 Center for Genomic Medicine, Desert Research Institute, Reno, NV 89502 USA; 26 Renown Institute for Cancer, Nevada System of Higher Education, Reno, NV 89502 USA; 27 The Donnelly Centre, University of Toronto, Toronto, Ontario Canada M5S 3E1 416-946-0863; 28 Virna Therapeutics, West Roxbury, MA USA; 29 Department of Microbiology Boston University, National Emerging Infectious Diseases Laboratories, Boston, MA 02118 USA; 30 Howard Hughes Medical Institute, New York, NY USA; 31 MBC, Department of Molecular Biotechnology and Health Sciences, University of Turin, Turin, TO 10126 Italy

**Keywords:** Infection, Medical genomics

## Abstract

SARS-CoV-2 is responsible for the ongoing world-wide pandemic which has already taken more than two million lives. Effective treatments are urgently needed. The enzymatic activity of the HECT-E3 ligase family members has been implicated in the cell egression phase of deadly RNA viruses such as Ebola through direct interaction of its VP40 Protein. Here we report that HECT-E3 ligase family members such as NEDD4 and WWP1 interact with and ubiquitylate the SARS-CoV-2 Spike protein. Furthermore, we find that HECT family members are overexpressed in primary samples derived from COVID-19 infected patients and COVID-19 mouse models. Importantly, rare germline activating variants in the *NEDD4* and *WWP1* genes are associated with severe COVID-19 cases. Critically, I3C, a natural NEDD4 and WWP1 inhibitor from *Brassicaceae*, displays potent antiviral effects and inhibits viral egression. In conclusion, we identify the HECT family members of E3 ligases as likely novel biomarkers for COVID-19, as well as new potential targets of therapeutic strategy easily testable in clinical trials in view of the established well-tolerated nature of the *Brassicaceae* natural compounds.

## Introduction

The Severe Acute Respiratory Syndrome Coronavirus 2 (SARS-CoV-2) associated with the emerging disease (COVID-19) has resulted in an unprecedented global health and economic crisis^1,2^. To date (March, 9, 2021), there are at least 24 putative drug treatments for the disease. However, most are still at early stages of research. The focus has been on the development of new and repositioned vaccines, monoclonal antibodies, and drugs^3–7^. Another treatment option involves passive antibody administration via convalescent plasma transfusion. Convalescent plasma has been successfully used in the past as post-exposure prophylaxis and in the therapeutic treatment of other coronavirus outbreaks (e.g., SARS-1 and Middle East respiratory syndrome [MERS]) and other autoimmune and chronic inflammatory diseases. Although some promising results have been initially reported^8^, no significant clinical efficacy has been documented in treated patients^9^. Several repurposed drugs have been tested with disappointing results and many other promising ones are undergoing clinical experimentation^10–13^. However, to date there is no effective specific target drug against COVID-19. The unavailability of selective and effective antiviral drugs is probably due to the poor knowledge of the pharmacological targets of the host cell necessary for the virus replication and/or for the egress of new virions. Thus, a deeper knowledge of SARS-CoV-2 virus-host interaction for is fundamental to understand the molecular mechanisms that underly the life cycle of COVID-19 in order to develop treatments worthy of a clinical trial assesement^14^.

The enzymatic activity of the HECT-E3 ligases has been implicated in the cell egression phase of some RNA viruses possibly highjacking the endosomal sorting complexes required for transport (ESCRT) machinery^15–17^, and specifically members of a subgroup of HECT-E3 ligases, known as C2-WW-HECT (NEDD4-like) comprising at least nine members in humans (NEDD4, NEDD4L, ITCH, SMURF1, SMURF2, WWP1, WWP2, HECW1, and HECW2). This subgroup is characterized by a common modular architecture composed of a C2 domain related to N-terminal C protein kinase, two to four domains with central tryptophan–tryptophan (WW), and a C-terminal HECT domain^18^. The C2 domain is a Ca^2+^-dependent binding domain and is mainly involved in targeting these enzymes to membrane compartments such as the plasma membrane, Golgi apparatus, endosomes, and lysosomes^18^. WW domains mediate protein– protein interactions through the recognition of Pro-rich motifs (PPxY, LPxY or related sequences) and phosphorylated Ser/Thr-Pro^19,20^. These domains provide a scaffold for recruiting protein substrates and regulators. Several viral proteins have been shown to recruit WW-domain host cell proteins of the NEDD4 family through PPxY motifs to facilitate their egression and diffusion^21,22^. Among them, WWP1 was found to interact with Ebola Virus VP40 to regulate egression suggesting that viral PPxY-host WW domain-mediated interaction could represent a potential new target for host-oriented inhibitors of EBOV and other virus egression^23^. Several studies have shown that the HECT family members not only physically interact with specific viral proteins to regulate the release of mature viral particles through the ESCRT machine, but to regulate endocytosis through ubiquitination^24^.

Here, we investigated the involvement of HECT family of E3 ligases in COVID-19 patients and their possible involvement in SARS-CoV-2 infection. We found that *WWP1*, *WWP2*, *SMURF1*, and *NEDD4* mRNA are overexpressed in COVID-19 vs. SARS-CoV-2 negative patients in nasopharyngeal and oropharyngeal swab cells, as well as in the lung of affected patients and in mouse models of COVID-19. We also identified a subset of rare allelic variants in these genes and studied their distribution in a large cohort of patients (COVID Human Genetic Effort (https://www.covidhge.com) severely affected by COVID-19 vs. asymptomatic or paucisymptomatic infected subjects. We showed that some of the identified variants display gain of function and aberrant activity. We finally evaluated whether selective inhibition of HECT proteins by a natural NEDD4 and WWP1 inhibitor from *Brassicaceae* displayed anti-SARS-CoV-2 activity, thus providing preclinical support for the possible development of clinical trials using this natural inhibitor in COVID-19 patients.

## Results

### HECT family members interact and ubiquitinate the SARS-CoV-2 Spike protein

**Fig. 1:**
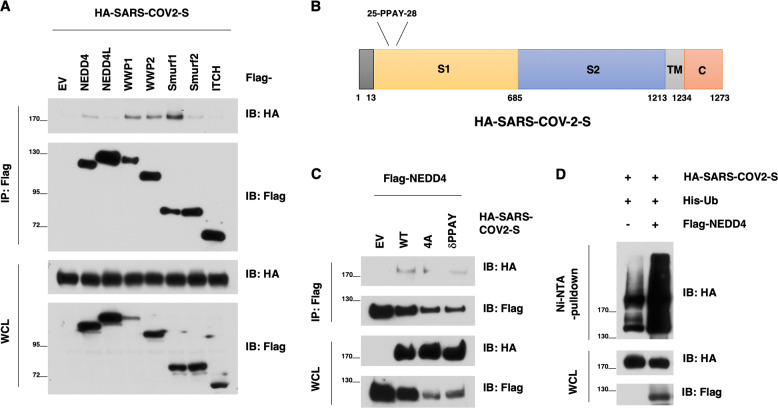
NEDD4 binds and ubiquitinates the SARS-CoV-2 S protein. **A** Immunoblotting (IB) of flag-immunoprecipates (IP) and whole cell lysis (WCL) derived from HEK293T cells that were transfected with HA-SARS-CoV-2 S and indicated NEDD4 family members. **B** A schematic diagram to show the PPxY motif in the SARS-CoV-2 S protein. **C** IB of flag-immunoprecipates and WCL derived from HEK293T cells that were transfected with NEDD4 and SARS-CoV-2 S WT or mutants. **D** IB of Ni-NTA pulldown and WCL derived from HEK293T cells that were transfected with SARS-CoV-2 S and NEDD4 WT (or EV as a negative control).

To determine whether and how HECT type of E3 ligase family members are involved in SARS-CoV-2 pathology, we at first focused on exploring how HECT E3 ligase might interact with the SARS-CoV-2 Spike protein, which plays a critical role for the virus infection and egression processes, and encode a PPxY motif (25-PPAY-28 in Spike protein)^25^. Notably, we found that the SARS-CoV-2 S protein could interact with several HECT-E3 family members, including NEDD4, WWP1, WWP2, SMURF1, and SMURF2 (Fig. 1A). Given that the PPxY motif is known to mediate the binding with NEDD4 family members^23^, we next mutated the PPAY motif to Alanine (4A) or deleted this motif (delta-PPAY) altogether (Fig. 1B). We found that these mutants reduced the binding with NEDD4 while not abrogating it entirely (Fig. 1C). Importantly and in support of a putative role of NEDD4 in regulating SARS-CoV-2 viral life cycle, we found that ectopic expression of NEDD4 could promote the ubiquitination of the SARS-CoV-2 S protein in cells (Fig. 1D).

### HECT genes and proteins expression in SARS-CoV-2 patients and mouse models

We first analyzed the gene expression levels of the nine HECT family members by qRT-PCR using specific primer pairs (Table 2) on cDNA from residual unidentified nasopharyngeal and oropharyngeal swabs of 37 COVID-19 patients with severe respiratory symptoms and 25 patients negative for the detection of SARS-CoV-2.

**Fig. 2:**
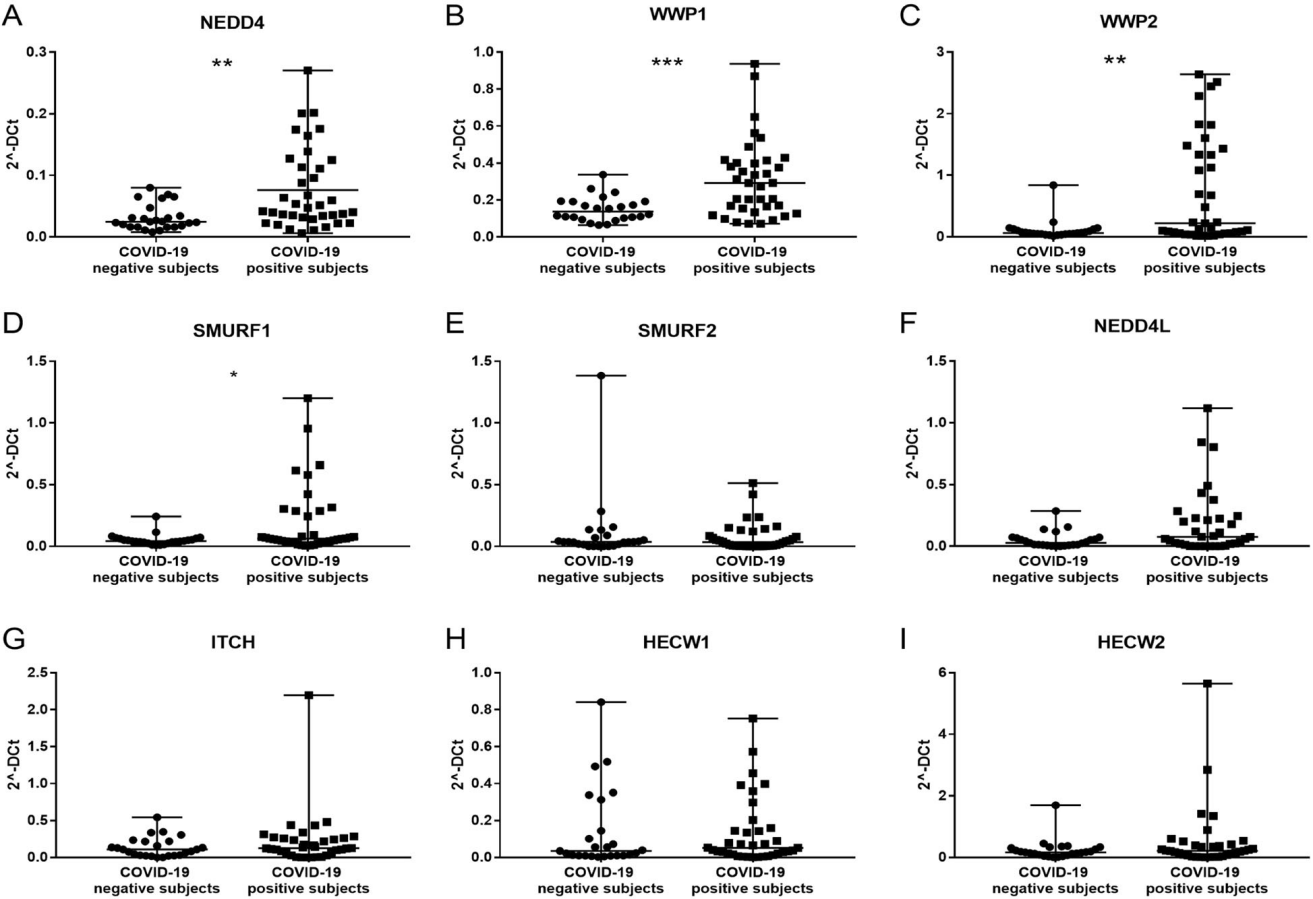
HECT E3 ubiquitin ligase gene expression level in SARS-CoV-2 positive and negative groups of subjects. **A** NEDD4 expression level in SARS-CoV-2 positive and negative groups of subjects, Mann–Whitney test, exact *p* value *p* = 0.0016, **; **B** WWP1 expression level in SARS-CoV-2 positive and negative groups of subjects, Mann–Whitney test, exact *p* value *p* = 0.0005, ***; **C** WWP2 expression level in SARS-CoV-2 positive and negative groups of subjects, Mann–Whitney test, exact *p* value *p* = 0.0038, **; **D** SMURF1 expression level in SARS-CoV-2 positive and negative groups of subjects, Mann–Whitney test, exact *p* value *p* = 0.044, *; **E** SMURF2 expression level in SARS-CoV-2 positive and negative groups of subjects, Mann–Whitney test, non-significant *p* value; **F** NEDD4L expression level in SARS-CoV-2 positive and negative groups of subjects, Mann–Whitney test, non-significant *p* value; **G** ITCH expression level in SARS-CoV-2 positive and negative groups of subjects, Mann–Whitney test, non-significant *p* value; **H** HECW1 expression level in SARS-CoV-2 positive and negative groups of subjects, Mann–Whitney test, non-significant *p* value; **I** HECW2 expression level in SARS-CoV-2 positive and negative groups of subjects, Mann–Whitney test, non-significant *p* value.

*NEDD4* (FC = + 2.06, *p* ≤ 0.005), *WWP1* (FC = + 1.85, *p* ≤ 0.0005), *WWP2* (FC = + 4.11; *p* < 0.005) and *SMURF1* (FC = + 1.7, *p* ≤ 0.05) showed a significant overexpression in nasopharyngeal and oropharyngeal swabs of COVID-19 positive patients compared to negative patients (Fig. 2A–D). No significant differences were observed in the other analyzed genes (Fig. 2E–I).

**Fig. 3:**
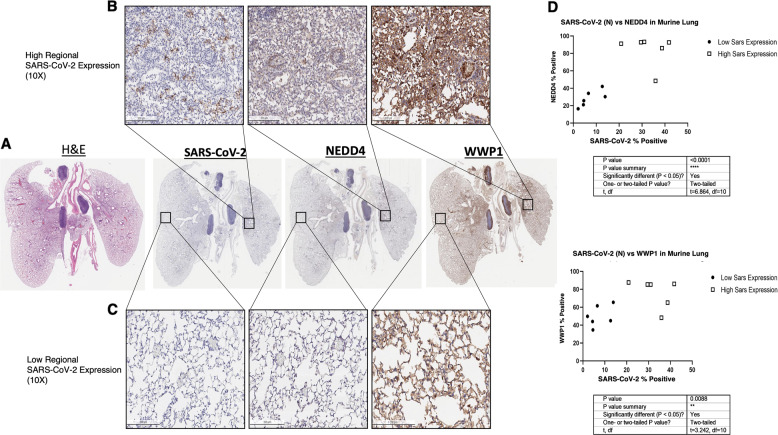
Mouse lungs (*n* = 3) expressing higher SARS-CoV-2 nucleocapsid protein express significantly higher NEDD4 (*p* < 0.0001*****) and WWP1 (*p* < 0.01**) in consecutive sections. Mouse lungs (*n* = 3) expressing higher SARS-CoV-2 nucleocapsid protein express significantly higher NEDD4 (*p* < 0.0001*****) and WWP1 (*p* < 0.01**) in consecutive sections (10X mag). Regions qualitatively defined as having high (>20% positive cells) Sars-CoV-2 expression (top row) and low (<20% positive cells) SARS-CoV-2 expression (bottom row), show with the corresponding regions in WWP1 and NEDD4 stained sections.

We then studied the expression of NEDD4 and WWP1 at protein level taking advantage of a COVID-19 mouse model and of available human lung specimens from infected patients necropsy. Overexpression at the protein level was observed for both WWP1 and NEDD4. However, WWP1 protein levels were more increased than those of NEDD4 in the SARS-CoV-2-infected human lung tissue. In PCR negative COVID-19 human lungs, the basal expression of NEDD4 and WWP1 was high. Interestingly, however, in PCR positive COVID-19 human lungs, NEDD4 and WWP1 were downregulated everywhere except in regions that expressed SARS-CoV-2 proteins (Supplementary Fig. 1). In keeping with the human data, both NEDD4 (*p* < 0,0001) and WWP1 (*p* < 0,01) proteins were significantly increased in mouse lungs overexpressing SARS-CoV-2 (Fig. 3). Thus, SARS-CoV-2 infection sustains and increases the expression levels of HECT Family members.

### HECT germline allelic variants in critical/life-threatening COVID-19 patients

We hypothesized that if high levels and sustained expression of specific HECT-E3 ligase family members are triggered by the SARS-CoV-2 infection, allelic variants that would affect their function could dictate the outcome and natural history of the disease. To test this hypothesis, we initially collected a cohort of 130 unrelated Italian SARS-CoV-2-positive patients, showing respiratory distress, Acute Respiratory Disease Syndrome (ARDS) or requiring invasive ventilation and Intensive Care Unit (ICU) admission. We identified a total of 408 HECT different pLOF, missense and in-frame monoallelic germline DNA variants. Data were extrapolated from previous studies performing WES analysis described in previous publications^26–28^. Introducing a cut-off at MAF < 0.01, we found 21 missense and 5 splice-region variants in *NEDD4*, *NEDD4L*, *SMURF1*, *SMURF2*, *HECW1*, *HECW2*, *WWP1*, and *WWP2* genes, in a total of 24 patients (Table 1). No variant was detected in the *ITCH* gene. The allelic frequencies of 12 genetic variants identified were significantly higher, when compared with those reported in GnomAD database for the EUR reference population (Table 1). One variant, M114I in *WWP2* gene, was never detected before (Table 1). Interestingly, five of the twelve variants observed with a higher frequency than that reported in GnomAD database are located in the *NEDD4* gene.

**Table 1:** HECT genes variants in a cohort of 130 SARS-CoV-2 positive patients (MAF < 0.01 in GnomAD v2.1.1; in bold *p* < 0.05).

Gene	Genetic form	Genotype	dbSNP	Consequence	AF GnomAD	Gender	Age [years]	*p*-value	Outcome
*HECW1*	Known	A1332T/WT	rs200973212	missense	0.0000161	M	54	**0.0114**	Survived
	Known	E502Q/WT	rs61756576	missense	0.0025630	M	83	0.5801	Deceased
	Known	N1265S/WT	rs200912368	missense	0.0017820	F	50	0.4656	Survived
*HECW2*	Known	S559G/WT	rs779373864	missense	0.0000085	M	59	**0.0072**	Survived
	Known	N417S/WT	rs138998510	missense	0.0005529	M	54	0.0791	Survived
	Known	A537P/WT	rs750339715	missense	0.0000121	F	93	**0.0092**	Deceased
*NEDD4*	Known	N888K/WT	rs759199057	missense	0.0000119	M	59	**0.01**	Survived
	Known	G451A/WT	rs60811367	missense	0.0017470	M	54	**0.0013**	Survived
						M	39		Deceased
	Known	I1237T/WT	rs373718024	missense	0.0003550	M	47	**0.0159**	Deceased
	Known	R877Q/WT	rs201295772	missense	0.0000958	M	83	**0.03176**	Deceased
	Known	I843R/WT	rs375088434	missense	0.0000199	F	77	**0.0091**	Deceased
	Known	T727I/WT	rs61754989	missense	0.0036830	F	72	1	Deceased
	Known	D129N/WT	rs150886795	missense	0.0002875	M	61	0.1233	Deceased
	Known	S29R/WT	rs115484917	missense	0.0026250	M	39	0.1555	Deceased
*NEDD4L*	Known	c.698C>T/WT	rs202231187	missense	0.0039100	M	73	1	Survived
	Known	c.1258-5A>C/WT	rs768158353	splicing	0.0000853	M	64	**0.0141**	Deceased
	Known	c.698C>T/WT	rs202231187	missense	0.0039100	M	54	1	Deceased
*SMURF1*	Known	R564Q/WT	rs182340234	missense	0.0000199	F	52	**0.0068**	Survived
	Known	T223M/WT	rs371859465	missense	0.0000805	F	80	**0.022**	Survived
*SMURF2*	Known	G10E/WT	rs866321574	missense	0.0061730	F	36	0.1166	Survived
						M	73		Deceased
	Known	I142V/WT	rs145845053	missense	0.0005564	M	14	0.1281	Survived
*WWP1*	Known	c.2395-4C>T/WT	rs188228045	splicing	0.0000040	F	89	1	Deceased
	Known	c.540-5T>C/WT	rs187132881	splicing	0.0023640	F	83	0.5073	Deceased
	Known	c.1836G>A/WT	rs150841032	splicing	0.0002012	M	76	0.1019	Deceased
*WWP2*	Known	R803C/WT	rs747018644	missense	0.0000043	M	54	**0.0049**	Survived
	New	M114I/WT	rs377573067	splicing	/	F	83	/	Deceased

In a second step, we extended the genetic study to an independent cohort of 710 unrelated COVID-19 critical patients and 483 controls with asymptomatic or mild SARS-CoV-2 infection belonging to the international CHGE Consortium data^29,30^, and we performed a PCA-adjusted burden test in order to evaluate a possible difference in the number of variants with MAF < 0.01. The analysis did not reveal an enrichment of pLOF/missense/inframe variants for any of the examined genes in severely affected patients when compared to the asymptomatic and paucisymptomatic infected controls (Supplementary Table 1). As those tests involved a large number of variants, it is likely that most of them are neutral and strongly decreased the power of this analysis by diluting the signal. Therefore, we performed a more detailed investigation of the variants that were present in at least two critical cases and absent in infected controls. We identified 13 variants among which, three of them emerged as deleterious in all in silico prediction tools (Supplementary Table 2). Two of the three identified deleterious variants were in *NEDD4* (I843R and R877G), and one in *WWP1* (N745S). Each of the three variants was present in two patients, and 3 out of the six patients carrying any of these variants died (Table 1, Supplementary Table 1). Interestingly, WWP1 has a known binding activity with the protein S of the virus, and the N745S missense variant previously characterized by Lee et al.^31^, leads to aberrant WWP1 enzymatic activation with subsequent PTEN inactivation, thereby triggering hyperactive growth-promoting PI3K signaling in cellular and murine models.

**Fig. 4:**
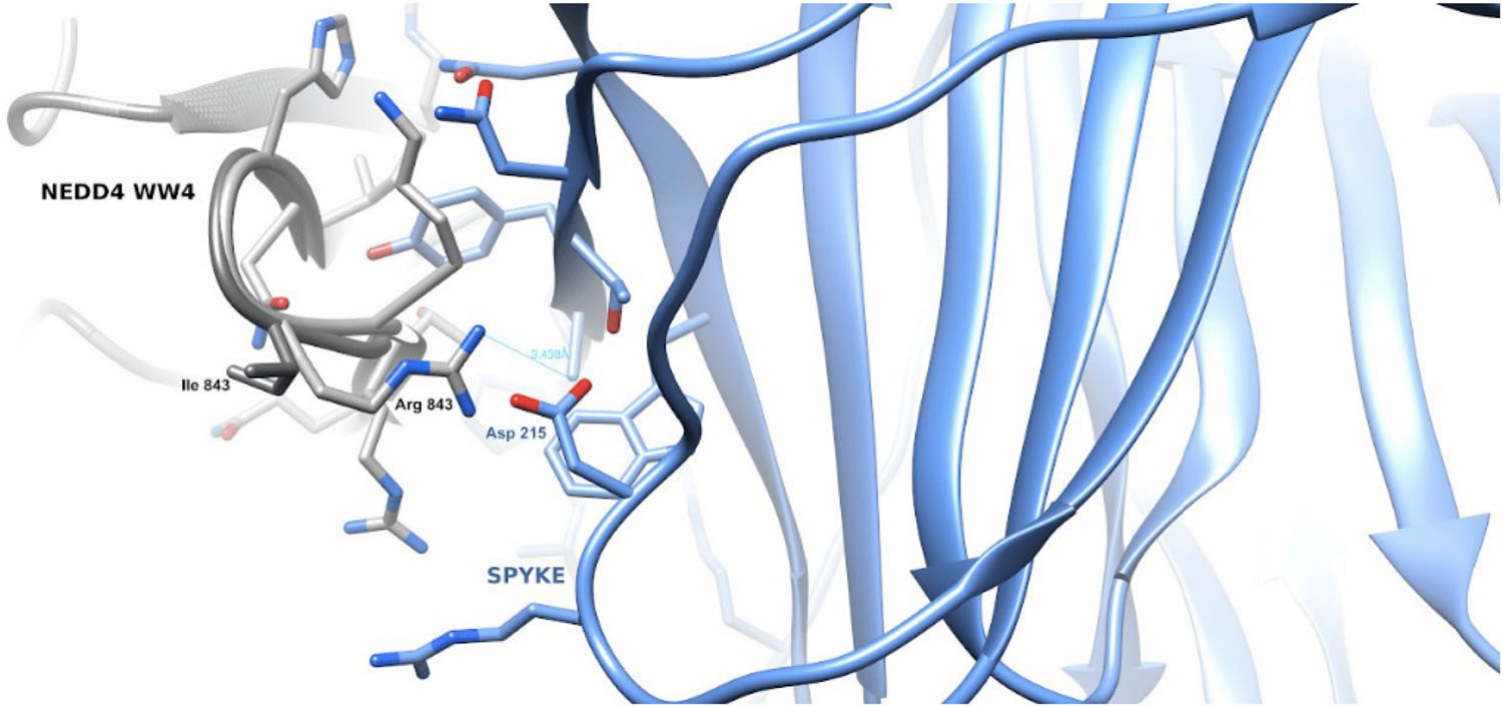
The Figure shows the 3D model of the WW domain of NEDD4 in complex with the SARS- CoV-2 Spike protein. The WW domain is displayed as a ribbon model, with the interface residue side-chains in gray. The Spike protein is displayed as a ribbon model, and the side-chains of its interface residues are shown in light blue. The Arg843–Asp215 residues are in close proximity and favor a stronger interaction between the variant WW domain and Spike with respect to the wt domain.

Next, we performed a more in depth in silico analysis of these three identified variants (I843R and R877G in *NEDD4*; N745S in *WWP1*). The I843R variant, mapping into the NEDD4 WW4 (in isoform 3), has a potential impact on the protein ability to interact with its substrates. Specifically, the 3D model of the variant WW4 domain in complex with the SARS-CoV-2 Spike (S) shows that the PPAY S residues interacting with the WW domain place the Asp215 S residue in close proximity with the Arg843 side-chain (Fig. 4), suggesting a stronger interaction between the two proteins compared to the WW4 domain wild type. The R877G variant maps between the WW4 and the HECTs domain of NEDD4 and most in silico methods for the evaluation of the impact of the variant (see Methods). The N745S variant in WWP1 maps inside its HECTc domain and seems not to affect the stability or the pathogenicity of the protein. Its position shows that the variation can influence the interaction between the HECT-type E3 ubiquitin transferase and its substrates. Furthermore, Lee et al.^31^ have demonstrated that this mutation can lead to an open and enzymatically active conformation of WWP1.

**Fig. 5:**
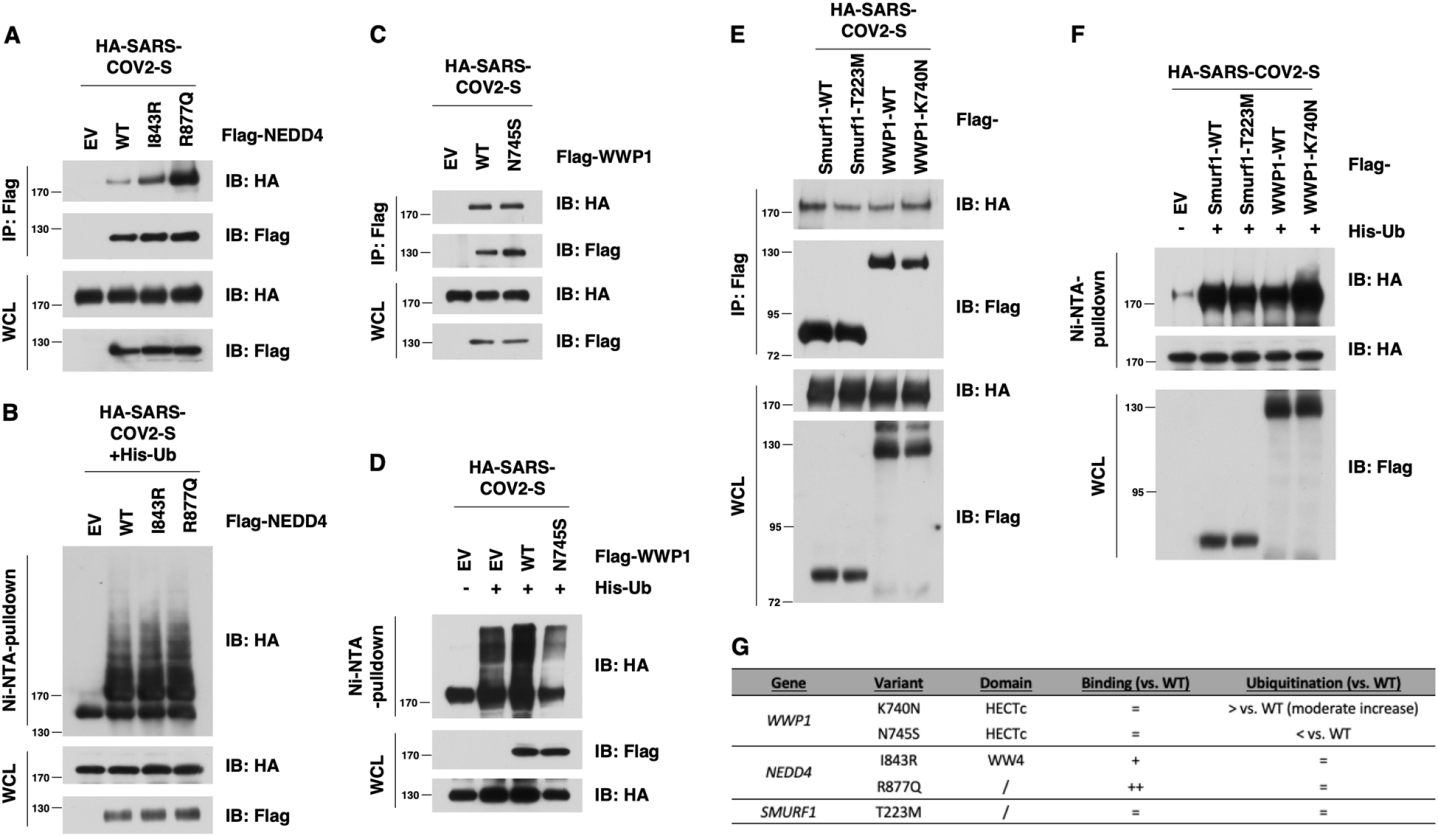
Gain-of-Function mutants in NEDD4, but not SMURF1 and WWP1, display elevated interaction with, but comparable ubiquitination of the SARS-CoV-2 S protein. **A**, **C** IB of flag-immunoprecipates and WCL derived from HEK293T cells that were transfected with HA-SARS-CoV-2 S and NEDD4/WWP1 WT and mutants. **B**, **D** IB of Ni-NTA pulldown and WCL derived from HEK293T cells that were transfected with SARS-CoV-2 S and NEDD4/WWP1 WT and mutants. **E** IB of flag-immunoprecipates and WCL derived from HEK293T cells that were transfected with HA-SARS-CoV-2 S and WT and mutants for Smurf1 or WWP1. **F** IB of Ni-NTA pulldown and WCL derived from HEK293T cells that were transfected with SARS-CoV-2 S and WT and mutants for Smurf1 or WWP1. **G** Binding and ubiquitination activity in WWP1/NEDD4/SMURF1 mutants vs. wild-type (WT).

To corroborate the in silico analysis, we next explored how these identified putative gain of function (GOF) mutations might impact the ability to interact or ubiquitinate the S protein. Notably, the two *NEDD4* variants derived from COVID-19 patients were able to more avidly bind with the SARS-CoV-2 Spike (S) protein compared to wt-NEDD4 (Fig. 5A, B). We also observed a slight increase in ubiquitination of the SARS-CoV-2 S protein in cells by the NEDD4 mutants (Fig. 5A, B), indicating that the GOF of NEDD4 might exert its regulatory function via direct interaction with the SARS-CoV-2 S protein and other S associated proteins. Similarly, we tested the K740N-WWP1 mutant, a gain of function mutant, implicated in cancer susceptibility as a positive control^31^. Once again, we observed increased binding and ubiquitination of the mutants over the WT control (Fig. 5E, F). However, despite the marked increased binding with Spike protein for the R877Q-NEDD4 mutant, we observed relatively comparable ability for WT-NEDD4 and R877Q-NEDD4 in promoting ubiquitination of the SARS-COV2 S protein in cells under this experimental setting (Fig. 5B). These results indicate that the NEDD4 hotspot mutations might exert its COVID-19 regulatory function via direct physiological interaction with the SARS-COV2 S protein and other S associated proteins, and the putative role of NEDD4-mediated ubiquitination of spike protein in COVID-19 biology awaits further in-depth studies. We also compared WWP1-WT versus WWP1 K740N and N745S, two germline variants that were implicated in cancer susceptibility and demonstrated to be gain-of-function mutation towards the tumor suppressor PTEN^31^. We utilized the WWP1 K740N mutant as a control for the WWP1 N745S COVID-19 associated mutant. We found that both WWP1 K750N and N745S mutants displayed comparable binding ability with SARS-CoV-2 S protein compared to WT-WWP1 (Fig. 5C–F). However, we observed a slight increase for K740N-mediated ubiquitination of the SARS-COV2 S protein, but a moderate decrease for N745S-mediated ubiquitylation of the SARS-COV2 S protein. These results argue that different WWP1 mutations might utilize different mechanisms to impact COVID-19 biology, which requires additional in-depth studies in the future. On the other hand, in comparison with the WT counterpart, the Smurf-1-T223M mutant exhibited comparable binding or ubiquitination ability on SARS-CoV-2 S protein (Fig. 5E, F), at least in this experimental setting. Given the close similarity between their biochemical features and the reported functional redundancies among NEDD4 family members, these data suggest that several NEDD4 family E3 ligases might participate in regulating COVID-19 egression via direct interaction with and ubiquitination of the SARS-CoV-2 S protein and associated proteins (Fig. 5G), but their potential complementary roles, as well as their biological and functional impacts in COVID-19 biology await further investigation.

### The HECT inhibitor I3C is effective in mediating SARS-CoV-2 antiviral effect in in vitro cellular models

**Fig. 6:**
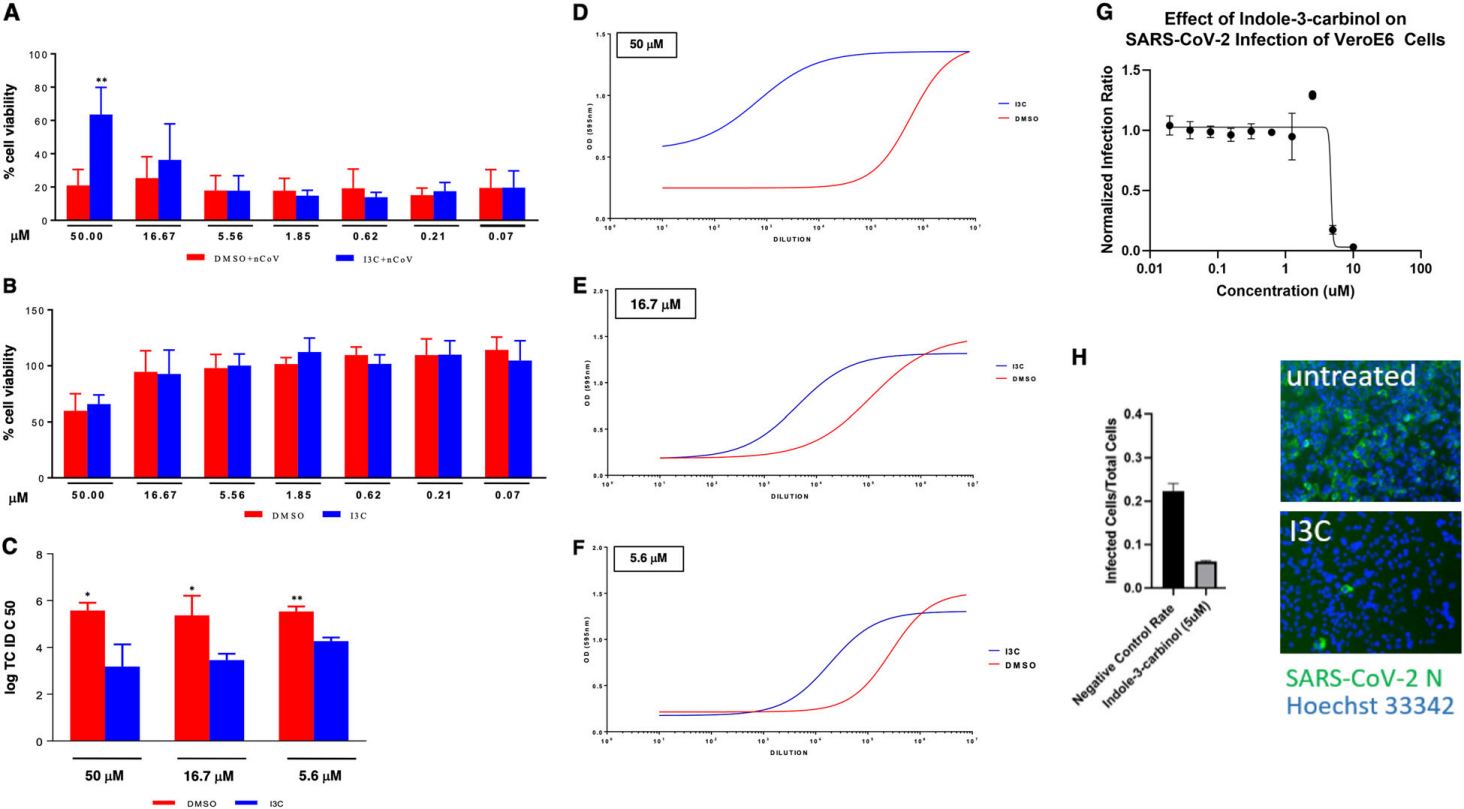
I3C inhibited SARS-CoV-2-induced CPE and viral production in Vero E6 cells. Cells were treated with different doses of I3C (from 50 to 0.069 μM; 1:3 serial dilutions) or DMSO (from 0.5 to 6.9 × 10^−4^ v/v percentage) 1 h before SARS-CoV-2 infection (MOI = 0.001) in four replicates. Absorption of the virus was allowed for 1 h at 37 °C in presence of I3C or DMSO treatments. The unabsorbed virus was removed and replaced by fresh medium with I3C or DMSO as above. Cells were then treated with either I3C or DMSO every 24 h and incubated at 37 °C with 5% CO_2_ for 72 h when the survival of infected (**A**) or not infected (**B**) cells was measured by crystal violet staining assay. The results were evaluated setting the uninfected control cells as 100% and the remaining values represented as a relative value. Experiments (*n* = 4) were performed in triplicate and data are expressed as mean +/−SD. Back-titration of virus progeny released by SARS-CoV-2-infected cells, treated as above, was performed on Vero E6 cells. Survival of the cells was measured by crystal violet staining assay. Results were analyzed using Graph Pad (GraphPad Prism 8 XML ProjecT) with nonlinear regression curve fit (Inhibitor vs. response-Variable slope (four parameters)) ((**D**–**F**) and data expressed as log TCID_50_/100 μl (**C**)). Statistically significant differences between DMSO and I3C are represented as *P < 0.05 or **P < 0.002 determined using the paired t-test. Vero E6 cells were challenged with SARS-CoV-2. After 1.5 d, cells were fixed, stained with an N specific antibody and Hoechst 33342 for cell nuclei. Infected cells and total cell nuclei were counted by image analysis. The infection efficiency was normalized to infection seen in vehicle (DMSO) treated cells (**G**, **H**).

We next hypothesized that if HECT-E3 ligases are indeed functionally relevant for the viral life cycle of COVID-19, Indole-3-carbinol (I3C), a natural NEDD4 and WWP1 inhibitor from *Brassicaceae*, might display a direct antiviral effect. We evaluated at first the impact of I3C on the cytopathic effect (CPE) induced by SARS-CoV-2 infection in Vero E6 cells. We treated the cells with I3C using a 3-fold concentration scale ranging between 50 and 0.069 μM. The drug was added at different time points, before (1 h) and after (1, 24, and 48 h) SARS-Cov-2 multiplicity of infection (MOI = 0.001). CPE was evaluated 72 h post-infection, when culture media were collected for viral titer measurement. We found that I3C reduced by about 60% the SARS-CoV-2-induced CPE in Vero E6 cells at 50 μM, when compared to DMSO-treated cells (Fig. 6A), while it was not effective at lower concentrations. Similar results were obtained using a 10-fold increased MOI at 48 h post-infection. To note that the concentration of 50 μM or 0.05% of I3C and DMSO, respectively, is partially toxic for the cells when treated for 72 h (Fig. 6B).

Importantly, however, a much greater effect was observed when assessing the impact of I3C treatment on the in vitro viral production. To this end, we measured the amount of infectious SARS-CoV-2 released by the infected cells treated with either I3C (50, 16.67, and 5.56 μM) or DMSO (0.5, 0.167 and 0.056% (v/v)). Notably, I3C significantly reduced the SARS-CoV-2 production at all the concentrations tested (Fig. 6C–F), with a virus yield reduction ranging from 2 to 4 log at the various I3C concentrations. The I3C-mediated decreased of viral production was also evident when cells were infected at higher MOI, although with lower efficacy (Supplementary Fig. 2). Since I3C reduced the viral production not only at 50 μM, when the CPE inhibition is clearly appreciated (Fig. 6D), but also at 16.67 and 5.56 μM, when SARS-CoV-2-induced CPE was not affected by I3C, it is likely that I3C reduced the viral release rather than a viral entry and/or replication leading to cell damage. Overall, these data demonstrated that I3C exerts a direct anti-SARS-CoV-2 replication activity.

We further tested the potential efficacy of I3C utilizing a inhibition assay in Vero cells (see “Methods”: “Virus infection inhibition assay”). Vero cells were grown to 70–90% confluency. After incubation with the virus cells were fixed in formalin and then stained with SARS-CoV-2 antibody against the N protein and a fluorescently tagged secondary antibody. Cell nuclei were stained with Hoechst 33342 dye. The cells were imaged using a Cytation (Biotek) automated imaging system to visualize the blue fluorescent nuclei and the green fluorescent infected cells expressing virus N protein. Images were analyzed by CellProfiler software using a customized analysis pipeline to count the nuclei and the infected cells. Infection efficiency is expressed as a function of infected cells/cell nuclei counted. Once again I3C was effective at inhibiting COVID-19 in this assay with an IC50 of 4.7 μm (Fig. 6G, H).

## Discussion

Several studies have shown that HECT proteins act as a functional interface between viral or cellular proteins containing PPxY motifs and the E-class vacuolar protein-sorting pathway (VPS)^32^. HECT domains can participate in specific protein-protein interactions^33^ and hence ubiquitination of substrate proteins, which appears necessary for PPxY-dependent viral budding. Among the HECT family members, WWP1 and NEDD4 have been the most implicated in PPxY motif-dependent viral budding, and their HECT ubiquitin ligase activity is required for this activity^34^. Critically, these two HECT ubiquitin ligases can physically and functionally interact forming heterodimeric complexes, and are druggable by a well-tolerated natural compound from Cruciferous vegetables^35^. Additionally, gain of function germ line mutations of WWP1 have been identified in cancer susceptibility syndromes and in cancer patients^31^.

We do not yet know the molecular mechanisms that govern several aspects of SARS-CoV-2 life cycle such as its entry, replication, assembly, budding, and particularly the egression of the virus. Recently, however, recently, Ghosh et al.^36^, using virus-specific imaging and reporter methodologies, demonstrated that ß-Coronaviruses utilize lysosomal trafficking for exit, rather than biosynthetic secretory pathway most commonly used by other enveloped viruses. The biochemical and molecular characterization of these steps and above all the identification of the proteins involved in these processes, is therefore crucial to develop drugs that could interfere and block fundamental processes in the biology of the virus and pave the way for new therapeutic approaches.

Based on in silico analysis, it was recently proposed that because SARS-CoV-2 encodes PPxY late domain motifs it might be capable of recruiting HECT family members and, therefore, the ESCRT complex to improve virus budding and release, favoring cellular reinfections. Interestingly, the PPxY motif is not present in SARS-CoV proteins. The presence of the motif PPxY might contribute to explain why SARS-CoV-2 is more contagious compared to SARS-CoV^22^.

Here we demonstrated that WWP1, WWP2, and NEDD4 are overexpressed during SARS-CoV-2 infection and that their expression co-localizes with areas of infection in lung tissue both in mice and humans. In addition, we also demonstrated that NEDD4 and WWP1, physically interact with and ubiquitylate the SARS-CoV-2 S protein. This demonstrates a direct involvement of the HECT family proteins and, in particular, of NEDD4 and WWP1 in the virus life cycle. It is therefore conceivable that a greater production or an increased enzymatic activity of members of these members of the HECT family could favor the exacerbation of the infection. The sole fact that they are overexpressed in concomitance with the SARS-CoV-2 infection suggests that the virus may take advantage from this pathway, as it has been shown for other RNA viruses.

Additionally, and in line with this notion, we identified three variants that bind more avidly to the SARS-CoV-2 S protein: two rare *NEDD4* variants (I843R and R877G) and the N745S germinal variant in *WWP1*, which was already characterized in cancer studies^31^. The increased binding affinity could favor the ubiquitination of viral and cellular proteins thus implementing vesicular packaging and virions release. These variants suggest the existence of a particular genetic constraint against loss of function or gain of function given the multifunctionality of HECT proteins^31^. It is worth noting that a recent paper reports that, in vitro, WWP1 K740 and 745S mutants displayed comparable ability as WT-WWP1 in largely monoubiquitinating PTEN. These mutants, therefore, do not appear to act as gain-of-function mutation in this in vitro setting^37^. On one hand, this report is consistent with what was previously reported in vivo where NEDD4 was found to mono-ubiquitinate PTEN and also cooperate with WWP1 in promoting K27-polyubiquitination of PTEN in cells, through heterodimeric interactions likely at plasma membrane^38^. On the other hand, the observed differences in WT-WWP1 vs. WWP1 mutants might stem from the monoubiquitination of PTEN observed in vitro ub assay vs. K27-polyubiquitination of PTEN detected in cells. It is possible that some key cellular factors, likely WWP1 interacting proteins, such as NEDD4, might be required for polyubiquitination of PTEN, both in cells and in vitro. Nonetheless, we also observed different ability for K740N and N745S WWP1 mutants in comparison with WT-WWP1 to promote ubiquitination of the SARS-CoV-2-S protein in cells (Fig. 5D, F). These results are in keeping with the Cole group^37^ to demonstrate that different WWP1 mutation might utilize different mechanism to control its downstream pathways, including PTEN and SARS-CoV-2 S protein, which warrants additional in-depth investigation to reveal the underlying complicated mechanism that is likely to be context dependent or ever unique to each individual mutation of WWP1. Further studies are warranted to analyze from a biochemical and functional point of view all the variants identified in these genes (Supplementary Table 1) in order to access the genetic enrichment found in a complete and unbiased way. It is also worth noting that several of the variants identified in these genes have been observed in both asymptomatic and critical subjects. Interestingly, we extended the genetic study to a second independent cohort of about 30,000 participants in the Healthy Nevada Project (HNP, Renown Health, Reno, Nevada, USA)^39^, to further corroborate our results. This analysis led to results comparable to the ones previously described. Moreover, we identified 9 additional rare variants never detected before in 9 COVID-19 patients, which may affect splicing (Supplementary Table 3).

HECT family members also play pivotal roles in the regulation of the innate immune response, and although the pathogenesis of COVID-19 is still under investigation, it is clear that the innate immunity plays a crucial role in protective or destructive responses upon SARS-CoV-2 infection^40^.

It is therefore conceivable that HEC family members may affect the outcome and natural history of the COVID-19 infection also impacting non-cell autonomous anti-viral defense mechanisms.

For instance, ITCH controls the stability of critical immune system proteins^41^ and acts upstream of B-cell lymphoma 6 (Bcl-6), the main transcription factor involved in coordinating follicular helper T-cell differentiation and immunoglobulin G (IgG) in response to acute viral infections^42^; WWP2 negatively regulates Toll-like receptor 3 (TLR3)-mediated innate immune response by targeting TIR-domain containing adapter-inducing interferon-β (TRIF) for ubiquitination and degradation^43^. The innate immune system acts as first responder for the detection and clearance of viral infections. Innate immune cells secrete proinflammatory cytokines that inhibit viral replication, stimulate the adaptive immune response, and recruit other immune cells to the site of infection^40^. In this respect, in an international cohort^29^, we recently showed that about 3% of COVID-19 critical patients carried loss-of-function variants in genes coding for proteins involved in type I IFN innate immunity, thus representing an important target for a deeper investigation of their role in the pathogenesis^30^. Collectively our results indicate that the risk of susceptibility to severe COVID-19 is unlikely to be influenced predominantly by rare variants of HECT genes in the MAF range <0,01. Rather, it is possible that rare (or private) variants may contribute substantially to the severity of the COVID-19 phenotype, suggesting that both very large sample sizes and gene-based association tests will be needed to carefully identify risk genetic factors.

While further studies will clarify the role and molecular mechanisms whereby HECT family members control the viral life cycle and the susceptibility and severity of COVID-19, our findings have immediate therapeutic implications for the treatment of infection and the prevention of the most severe outcomes triggered by the virus. The fact that I3C is effective in reducing SARS-CoV-2 production in vitro prompts the immediate assessment of its efficacy in clinical trials (Supplementary Fig. 3). I3C is, in fact, well-tolerated in both animal models and phase I trials in humans at doses effective in the in vitro cell models^35,44^. It is therefore conceivable to rapidly reposition I3C in Phase II clinical trials in humans to test its ability to prevent the clinical severity of COVID-19.

## Materials and methods

### International CHGE Consortium database

Between March and April 2020, 130 patients with COVID-19 diagnosis were enrolled on Protocol no. 50/20 (Tor Vergata University Hospital). Informed consent was obtained from each patient.

To further improve our cohort, other 710 cases and 483 controls were enrolled from the COVID Human Genetic Effort, International CHGE Consortium (Casanova J.L. and Su H., https://www.covidhge.com), as described in Zhang et al.^30^.

The institutional review boards of each participating Institution approved the protocol prior to patient enrollment. The study was conducted in agreement with the principles of Declaration of Helsinki.

### Whole exome sequencing and data pre-processing

Genomic DNA was extracted from peripheral blood samples using standard procedures and Qiagen blood DNA mini Kit (Qiagen, Hilden, Germany). Library preparation and whole exome capture were performed by using the Twist Human Core Exome Kit (Twist Bioscience, South San Francisco, CA, USA) according to the manufacture’s protocol and sequenced on the Illumina NovaSeq 6000 platform. The BaseSpace pipeline (Illumina, Inc., San Diego, CA, USA) and the TGex software (LifeMap Sciences, Inc., Alameda, CA, USA) were used for the variant calling and annotating variants, respectively. Sequencing data were aligned to the hg19 human reference genome. A minimum depth coverage of 30X was considered suitable for analysis, based on the guidelines of the American College of Medical Genetics and Genomics. All variants were examined for coverage and Qscore (minimum threshold of 30) and visualized by the Integrative Genome Viewer (IGV).

### Gene expression

#### Patients’ recruitment and sample collection for HECT3 ligase family expression study

During the months of March and April 2020, we collected the nasopharyngeal and oropharyngeal swabs of 62 subjects with acute respiratory symptoms or contacts with COVID-19 confirmed cases, arrived at the attention of the Emergency Room (ER) of Policlinico Tor Vergata, PTV (Rome, Italy). As widely described by Amati et al.^45^, patients’ swabs were referred to the Virology Unit of PTV for the molecular diagnostic test detecting the presence of SARS-CoV-2 nucleic acids using used the Allplex™ 2019-nCoV Assay (Seegene Inc, http://www.seegene.com/upload/product/Allplex_2019_nCoV_performance_data.pdf).

SARS-CoV-2 positive (*n* = 37) and negative (*n* = 25) samples were used for RNA expression analysis.

#### Real-time PCR and statistical analysis

The total RNA extracted from nasopharyngeal and oropharyngeal swabs was evaluated by NanoDrop DS-11 (DeNovix) and 100 ng of total RNA was been reverse transcribed into cDNA using the High Capacity cDNA Reverse Transcription Kit (Applied Biosystems, USA). We analyzed the expression of the 9 members of the HECT3 ligase family: *WWP1*, *WWP2, NEDD4*, *NEDD4L, ITCH, SMURF1, SMURF2, HECW1*, and *HECW2* genes; *GAPDH, ACTB*, and *RPLP0* genes were used for data normalization. Real-time PCRs (qRT-PCRs) have been performed using ABI7500 Fast Real-time PCR System (Life Technologies) with Sybr Green Assay (Power Sybr Green PCR Master Mix, Life Technologies) and specific primer pairs (Table 2).

**Table 2:** Real-Time PCR primer sequences.

Gene	Accession number	Sequence (5’→3’)		Product size (bp)
WWP1	NM_007013.4	Fw	TGTAAATGTTACGCCACAGACT	105
		Rv	GCTTGTTTCAAATCTATCGTTGC	
WWP2	NM_007014.5	Fw	GAAAGTGGTGTCCGCAAAGC	175
		Rv	ATGACTCTGTGCCGTGACATT	
NEDD4	NM_006154.4	Fw	CTGCTACGGACAATTATACCCTA	129
		Rv	CATCCAACAGTTTGCCATGATA	
NEDD4L	NM_001144967.3	Fw	ACGTAGCGGATGAGAATAGAGAAC	115
		Rv	CTGTGATTAGATGGGTTTACCCTGA	
ITCH	NM_031483.7	Fw	GGTTCAGTATTTCCGGTTCTGGT	118
		Rv	GGGACTGAAGCTCATTATCTGTTG	
SMURF1	NM_020429.3	Fw	CCGCTCCAAGGCTTCAAGG	125
		Rv	ATCCGGTTAAAGCAGGTATGGG	
SMURF2	NM_022739.4	Fw	GCAAATGGATCAGGAAGTCGGAAA	100
		Rv	CCGGAGGCCGGAGGA	
HECW1	NM_015052.5	Fw	CGAGCAACCACCCCCAGTGT	136
		Rv	CCATGGCTTGGAAATCTGAGAGA	
HECW2	NM_001348768.2	Fw	CTACCAGCATAACCGCGACC	112
		Rv	AAAGAATGCCTTGCCCTGGT	
GAPDH	NM_002046	Fw	AAGGTCGGAGTCAACGGATTT	100
		Rv	TGAAGGGGTCATTGATGGCA	
ACTB	NM_001101	Fw	ATTGCCGACAGGATGCAGAA	150
		Rv	GCTGATCCACATCTGCTGGAA	
RPLP0	NM_001002	Fw	ACCCAGCTCTGGAGAAACT	198
		Rv	AAAAGGAGGTCTTCTCGGG	

*WWP1* WW domain containing E3 ubiquitin protein ligase 1, *WWP2* WW domain containing E3 ubiquitin protein ligase 2, *NEDD4* neural precursor cell expressed, developmentally downregulated 4, E3 ubiquitin protein ligase, *NEDD4L* neural precursor cell expressed, developmentally downregulated 4-like, E3 ubiquitin protein ligase, *ITCH* HECT-type E3 ubiquitin transferase itchy homolog, *SMURF1* SMAD specific E3 ubiquitin protein ligase 1, *SMURF2* SMAD specific E3 ubiquitin protein ligase 2, *HECW1* HECT, C2 and WW domain containing E3 ubiquitin protein ligase 1, *HECW2* HECT, C2 and WW domain containing E3 ubiquitin protein ligase 2, *GAPDH* glyceraldehyde-3-phosphate dehydrogenase, *ACTB* *β-actin*, *RPLP0* ribosomal protein, large, P0, *Fw* forward, *Rev* reverse. *PCR* polymerase chain reaction.

The qRT-PCR expression analyses were performed in triplicate. Data analysis was performed using the comparative threshold cycle (Ct) method quantification (2^−ΔCt^ method) (as described by Rizzacasa et al. at https://www.protocols.io/view/comparative-ct-method-quantification-2-ct-method-zp7f5rn).

Statistical analysis was performed using GraphPad Prism 7.0 (GraphPad Software, USA). D’Agostino & Pearson, Shapiro–Wilk, and Kolmogorov–Smirnov normality tests were used to assess the distribution of gene expression data derived from qRT-PCR assays. Since gene expression data did not pass the normality test (*p* ≤ 0.05), Mann–Whitney test was used for data comparison between SARS-CoV-2 positive and negative groups. In graphs, gene expression is represented as median with range. Significance was set at a minimum of *p* ≤ 0.05.

### Histology and Immunohistochemistry

Autopsy tissue was sourced from patients (*n* = 3) at the Harris County Institute of Forensic Sciences and Memorial Herman Hospital, Texas Medical Center, who tested positive for SARS-CoV-2 by nasopharyngeal swab RT-PCR. Autopsy lungs were removed and fixed in 10% neutral buffered formalin for 24 h. BALB/c mice (*n* = 3) were transduced with 2.5e8 PFUs of AdV-hACE2. Then, 5 days later, mice were infected with SARS-CoV-2 and tissues were collected from day 4 post infection. Murine lungs were fixed in 10% neutral buffered formalin for 48 h. All samples were processed, paraffin embedded and sectioned at 4 μm at HistoWiz (histowiz.com).

Immunohistochemistry was performed by HistoWiz using Bond Polymer Refine Detection Kit (Leica Biosystems) and Leica Bond Rx automated stainer (Leica Biosystems) with the following antibodies: rabbit polyclonal anti-SARS-CoV-2 Nucleocapsid (N) protein (Novus Biologicals 100-56576, 0.5 mg/ml), rabbit polyclonal anti-human NEDD4 (EMD Millipore 07-049, 1 mg/ml), and mouse monoclonal (1A7) anti-human WWP1 (Abnova H00011059-M01, 0.32 mg/ml). Slides were coverslipped using a TissueTek-Prisma film coverslipper (Sakura). Whole slide scanning (40x) was performed on an Aperio AT2 (Leica Biosystems).

Animal work was conducted adhering to the institution’s guidelines for animal use, and followed the guidelines and basic principles in the United States Public Health Service Policy on Humane Care and for Use of Laboratory Animals, and the Guide for the Care and Use of Laboratory Animals by certified staff in an Association for Assessment and Accreditation of Laboratory Animal Care (AAALAC) International accredited facility (protocol n. IACUC-2014-0255).

#### Image analysis and statistical analysis

Whole slide svs images of the three different stains were aligned to one another using the automated image alignment QuPath software (https://qupath.github.io), to permit the identification of similar regions within the tissue section between separate stains. Digital image analysis was performed using Definiens TissueStudio 4.0 software (AstraZeneca, Munich Germany), in which nucleus detection was performed on the hematoxylin counterstain, and positive cells were identified using a threshold for DAB staining to identify positive stained images for each biomarker (NEDD4, WWP1, and SARS-CoV2 Nucleocapsid protein).

Following quantification, expression (as % positive cells) of NEDD4 and WWP1 was plotted against SARS-CoV2 expression using Prism software. Six random regions of high SARS-CoV2 expression (>20% positive cells) and 6 random regions low SARS-CoV-2 expressions (<20% positive cells) were identified, and expression of NEDD4 and WWP1 within these regions were quantified. Two-tailed t-tests were performed to compare expression of NEDD4 and WWP1 between the high and low SARS-CoV-2 expression groups.

### In silico analysis

For the NEDD4 and WWP1 proteins the evaluation of the probability that the selected variants can have an impact on the protein function has been extracted from the Ensembl genome browser^46^, relying on the SIFT, PolyPhen, CADD, REVEL, MetalR, and Mutation Assessor methods^46–51^. For the evaluation of the Ile843Arg NEDD4 variant, we built the 3D model of the fourth WW domain of isoform 3 using the 1I5H PDB structure as template (displaying 87% sequence identity with our query^52^). The chosen template corresponds to the fourth WW domain of rat NEDD4 protein. The reconstruction of the possible complex between the human WW4 domain of NEDD4 and the Spike protein of SARS-CoV-2 was performed using the 4N7H PDB complex formed by the WW3 of human NEDD4 protein in complex with its bound peptide, characterized by the typical PPxY WW binding sequence^53^. The 3D model of the WW4 domain of NEDD4 was superposed on the Cα and Cβ atoms of the complex interface, while residues 24–28 of the Spike protein (PDB: 6XR8 were superposed on the corresponding PPxY sequence of the 4N7H peptide^54^. The complex model was subsequently assessed with 100 cycles of steepest descent minimization and evaluated with the Chimera v1.14 software^55^.

### Functional experiments

#### Immunoblots and co-immunoprecipitation experiments

Immunoblots and co-immunoprecipitation analysis of COVID-19 Spike and HECT-family members (wt vs. mutants) were performed in HEK293T cells as previously described^30,31^, and as described in the legends to Figs. 1 and 5.

#### HECT inhibitor I3C: cells

Vero E6 cells are kidney epithelial cells originally extracted from an African green monkey (Chlorocebus sp.; formerly called Cercopithecus aethiops). Cells were maintained in Minimum Essential Medium (MEM), supplemented with heat inactivated 10% fetal bovine serum (FBS), 2 mM L-glutamine and 1% penicillin/streptomycin solution (Sigma-Aldrich, Cat.No. R0883; F7524; G7513; P0781, respectively) and maintained at 5% CO_2_, 37 °C.

#### I3C antiviral test

The antiviral activity of I3C has been tested by a cytopathic effect (CPE) inhibition assay using Vero E6 cells infected with the SARS-CoV-2 strain isolated at INMI L. Spallanzani IRCCS (2019-nCoV/Italy-INMI1; GenBank MT066156^56^). The extent of in vitro inhibition of SARS-CoV-2-driven cell damage (CPE) by I3C is expressed as percentage of surviving cells.

Briefly, cell monolayers growing in 96-well plates (3 × 10^4^ cells/well) were treated for 1 h with 1:3 serial dilutions of I3C before SARS-CoV-2 infection. DMSO was used as uninfected control since I3C is solubilized in this compound. Cells were infected at MOI = 0.001 using MEM supplemented with heat inactivated 2% FBS and 2 mM L-glutamine in the presence of I3C/DMSO treatments. After 1 h of incubation the viral input was replaced by fresh medium containing either I3C or DMSO. Cells were then treated with either I3C or DMSO every 24 h and incubated at 37 °C with 5% CO_2_ for 72 h, when cell viability was measured by a standard crystal violet staining assay, measuring the optical density (OD) at 595 nM. Results were analyzed using Graph Pad (GraphPad Prism 8 XML ProjecT) and reported as the percentage of survived cells respect to the not-infected cells.

#### SARS-CoV-2 yield reduction assay

SARS-CoV-2 progeny released in culture medium during the antiviral assays was back-titrated by CPE assay on Vero E6 cells. The media of I3C- or DMSO-treated SARS-CoV-2-infected cultures were serially diluted in four replicates using MEM supplemented with 2% FCS, 2 mM L-glutamine, loaded on Vero E6-containing 96-well plates (3 × 10^4^ cells/well), and incubated at 37 °C for 72 h; CPE was measured by a standard crystal violet staining as described above. Results were analyzed using Graph Pad (GraphPad Prism 8 XML ProjecT) with nonlinear regression curve fit (Inhibitor vs. response-variable slope (four parameters)) and virus titres expressed as log tissue culture infectious dose (TCIC)_50_/100 μl.

#### Virus Infection inhibition assay

Vero E6 cells were obtained from ATCC (Manassas, VA, USA) and grown in DMEM with 10% fetal bovine serum (FBS) at 37 °C. The virus strain utilized was isolated from a traveler returning to Washington State, USA from Wuhan, China (USA-WA1/2020) and was obtained from BEI resources (Manassas, VA, USA). The virus stock was passaged twice on Vero E6 cells by challenging at an MOI of less than 0.01 and incubating until cytopathology was seen (after about 3 days). A sample of the culture supernatant was sequenced by NGS and was consistent with the original isolate without evidence of contaminants. The virus stock was stored at −80 °C until used.

For evaluation of indole-3-carbinol against infection with wild type SARS-CoV-2, the compound was dissolved to 10 mM in DMSO and then diluted in culture medium before addition to cells. The compound was added to VeroE6 cells incubated for a minimum of 1 hour, then challenged with virus at an MOI of less than 0.2. Dosing ranged from a final concentration of 10 µM down to 0.02 µM in a two-fold dilution series. As a positive control, 5 µM E-64 was used as it was previously reported to inhibit SARS-CoV-2 infection^57^. Negative controls were <0.5% DMSO. After a ~1.5 day incubation, cells were treated with 10% buffered formalin for at least 6 h, washed in PBS and virus antigen stained with SARS-CoV-2 specific antibody (Sino Biologicals, MM05) together with Hoechst 33342 dye to stain cell nuclei. Plates were imaged by a Biotek Cytation 1 microscope and automated image analysis was used to count total number of infected cells and total cell nuclei. CellProfiler software (Broad Institute, MA, USA) was used for image analysis using a customized processing pipeline (available upon request to RAD). Infection efficiency was calculated as the ratio of infected cells to total cell nuclei, and treatment conditions were normalized to the average of the negative controls. Loss of cell nuclei was used to flag treatments suggestive of toxicity. IC50 value was calculated using dose-response models fitted by GraphPad Prism software. The assay was performed in duplicate in 384 well plates.

### Supplementary information

Supplementary Table 1

Supplementary Table 2

Supplementary Table 3

Supplementary Figure 1

Supplementary Figure 2

Supplementary Figure 3

Related Manuscript File

Related Manuscript File

Supplementary Figure legends

## Data Availability

The datasets used and/or analyzed during the current study are available from the corresponding author on reasonable request. Additional supporting information may be found in the online version of this article at the publisher’s web site.

## Appendix I: COVID Human Genetic Effort

Laurent Abel^1^, Alessandro Aiuti^2^, Saleh Al-Muhsen^3^, Fahd Al-Mulla^4^, Mark S. Anderson^5^, Andrés Augusto Arias^6^, Hagit Baris Feldman^7^, Dusan Bogunovic^8^, Alexandre Bolze^9^, Anastasiia Bondarenko^10^, Ahmed A. Bousfiha^11^, Petter Brodin^12^, Yenan Bryceson^13^, Carlos D. Bustamante^14^, Manish J. Butte^15^, Giorgio Casari^16^, Samya Chakravorty^17^, John Christodoulou^18^, Antonio Condino-Neto^19^, Stefan M. Constantinescu^20^, Megan A. Cooper^21^, Clifton L. Dalgard^22^, Murkesh Desai^23^, Beth A. Drolet^24^, Sara Espinosa-Padilla^25^, Jacques Fellay^26^, Carlos Flores^27^, José Luis Franco^6^, Antoine Froidure^28^, Peter K. Gregersen^29^, Filomeen Haerynck^30^, David Hagin^31^, Rabih Halwani^32^, Lennart Hammarström^33^, Jim Heath^34^, Sarah E. Henrickson^35^, Elena Hsieh^36^, Kohsuke Imai^37^, Yuval Itan^38^, Timokratis Karamitros^39^, Kai Kisand^40^, Cheng-Lung Ku^41^, Yu-Lung Lau^42^, Yun Ling^43^, Carrie L. Lucas^44^, Tom Maniatis^45^, Davoud Mansouri^46^, László Maródi^47^, Isabelle Meyts^48^, Joshua D. Milner^49^, Kristina Mironska^50^, Trine H. Mogensen^51^, Tomohiro Morio^52^, Lisa F.P. Ng^53^, Luigi D. Notarangelo^54^, Antonio Novelli^55^, Giuseppe Novelli^56^, Satoshi Okada^57^, Tayfun Ozcelik^58^, Jana M. Pachlopnik^59^, Qiang Pan-Hammarström^60^, Rebeca Perez de Diaz^61^, Anna M. Planas^62^, Carolina Prando^63^, Aurora Pujol^64^, Lluis Quintana-Murci^65^, Laurent Renia^53^, Carlos Rodríguez-Gallego^66^, Vanessa Sancho-Shimizu^67^, Vijay Sankaran^68^, Mohammed Shahrooei^69^, Andrew L. Snow^22^, Pere Soler-Palacín^70^, András N. Spaan^71^, Stuart G. Tangye^72^, Stuart Turvey^73^, Furkan Uddin^74^, Mohammed J. Uddin^75^, Diederik van de Beek^76^, Donald C. Vinh^77^, Horst von Bernuth^78^, Pawel Zawadzki^79^, Helen C. Su^54*^, Jean-Laurent Casanova^80*^

^1^INSERM U1163, University of Paris, Imagine Institute, Paris, France. ^2^San Raffaele Telethon Institute for Gene Therapy, IRCCS Ospedale San Raffaele, and Vita Salute San Raffaele University, Milan, Italy. ^3^Immunology Research Laboratory, Department of Pediatrics, College of Medicine and King Saud University Medical City, King Saud University, Riyadh, Saudi Arabia. ^4^Dasman Diabetes Institute, Department of Genetics and Bioinformatics, Dasman, Kuwait. ^5^Diabetes Center, University of California, San Francisco, San Francisco, CA, USA. ^6^Universidad de Antioquia, Group of Primary Immunodeficiencies, Antioquia, Colombia. ^7^The Genetics Institute, Tel Aviv Sourasky Medical Center and Sackler Faculty of Medicine, Tel Aviv University, Tel Aviv, Israel. ^8^Icahn School of Medicine at Mount Sinai, New York, NY, USA. ^9^Helix, San Mateo, CA, USA. ^10^Shupyk National Medical Academy for Postgraduate Education, Kiev, Ukraine. ^11^Clinical Immunology Unit, Department of Pediatric Infectious Disease, CHU Ibn Rushd and LICIA, Laboratoire d’Immunologie Clinique, Inflammation et Allergie, Faculty of Medicine and Pharmacy, Hassan II University, Casablanca, Morocco. ^12^SciLifeLab, Department of Women’s and Children’s Health, Karolinska Institutet, Stockholm, Sweden. ^13^Department of Medicine, Center for Hematology and Regenerative Medicine, Karolinska Institutet, Stockholm, Sweden. ^14^Stanford University, Stanford, CA, USA. ^15^Division of Immunology, Allergy, and Rheumatology, Department of Pediatrics, UCLA, Los Angeles, CA, USA; Department of Microbiology, Immunology, and Molecular Genetics, UCLA, Los Angeles, CA, USA. ^16^Medical Genetics, IRCCS Ospedale San Raffaele, Milan, Italy. ^17^Department of Pediatrics and Children’s Healthcare of Atlanta, Emory University, Atlanta, GA, USA. ^18^Murdoch Children’s Research Institute, Victoria, Australia. ^19^Department of Immunology - Institute of Biomedical Sciences - University of São Paulo, São, Brazil. ^20^Endocrinology Department, Cliniques Universitaires Saint Luc, Brussels, Belgium. ^21^Washington University School of Medicine, St. Louis, MO, USA. ^22^Department of Anatomy, Physiology & Genetics, Uniformed Services University of the Health Sciences, Bethesda, MD, USA. ^23^Bai Jerbai Wadia Hospital for Children, Mumbai, India. ^24^School of Medicine and Public Health, University of Wisconsin, Madison, WI, USA. ^25^Instituto Nacional de Pediatria (National Institute of Pediatrics), Mexico City, Mexico. ^26^School of Life Sciences, Ecole Polytechnique Fédérale de Lausanne, Lausanne, Switzerland; Precision Medicine Unit, Lausanne University Hospital and University of Lausanne, Lausanne, Switzerland. ^27^Genomics Division, Instituto Tecnológico y de Energías Renovables (ITER), Santa Cruz de Tenerife, Spain; Research Unit, Hospital Universitario N.S. de Candelaria, Santa Cruz de Tenerife, Spain; Instituto de Tecnologías Biomédicas (ITB), Universidad de La Laguna, San Cristóbal de La Laguna, Spain; CIBER de Enfermedades Respiratorias, Instituto de Salud Carlos III, Madrid, Spain. ^28^Institut de Recherche Expérimentale et Clinique, Pôle de Pneumologie, Université catholique de Louvain, Belgium Service de pneumologie, Cliniques Universitaires Saint-Luc, Brussels, Belgium. ^29^Feinstein Institute for Medical Research, Northwell Health USA, Manhasset, NY, USA. ^30^Department of Paediatric Immunology and Pulmonology, Centre for Primary Immunodeficiency Ghent (CPIG), PID Research Laboratory, Jeffrey Modell Diagnosis and Research Centre, Ghent University Hospital, Edegem, Belgium. ^31^The Genetics Institute Tel Aviv Sourasky Medical Center, Tel Aviv, Israel. ^32^Sharjah Institute of Medical Research, College of Medicine, University of Sharjah, Sharjah, United Arab Emirates. ^33^Department of Laboratory Medicine, Karolinska Institutet, Stockholm, Sweden. ^34^Institute for Systems Biology, Seattle, WA, USA. ^35^Perelman School of Medicine, University of Pennsylvania Children’s Hospital of Philadelphia, Division of Allergy-Immunology, Philadelphia, PA, USA. ^36^Department of Immunology and Microbiology, Department of Pediatrics, Division of Allergy and Immunology, University of Colorado, School of Medicine Children’s Hospital Colorado, Aurora, CO, USA. ^37^Riken, Tokyo, Japan. ^38^Institute for Personalized Medicine, Icahn School of Medicine at Mount Sinai, New York, NY, USA; Department of Genetics and Genomic Sciences, Icahn School of Medicine at Mount Sinai, New York, NY, USA. ^39^Bioinformatics and Applied Genomics Unit, Department of Microbiology, Hellenic Pasteur Institute, Athens, Greece. ^40^Molecular Pathology, Department of Biomedicine, Institute of Biomedicine and Translational Medicine, University of Tartu, Tartu Estonia. ^41^Chang Gung University, Taoyuan County, Taiwan. ^42^Department of Paediatrics & Adolescent Medicine, The University of Hong Kong, Hong Kong, China. ^43^Shanghai Public Health Clinical Center, Fudan University, Shanghai, China. ^44^Department of Immunobiology, Yale University School of Medicine, New Haven, CT, USA. ^45^Zukerman Mind Brain Behavior Institute, Columbia University, New York, NY, USA; New York Genome Center, New York, NY, USA. ^46^Department of Clinical Immunology and Infectious Diseases, National Research Institute of Tuberculosis and Lung Diseases, The Clinical Tuberculosis and Epidemiology Research Center, National Research Institute of Tuberculosis and Lung Diseases (NRITLD), Masih Daneshvari Hospital, Shahid Beheshti University of Medical Sciences, Tehran, Iran. ^47^PID Clinical Unit and Laboratory, Department of Dermatology, Venereology and Dermato-oncology. ^48^Department of Pediatrics, University Hospitals Leuven, Leuven, Belgium; Laboratory for Inborn Errors of Immunity, KU Leuven, Leuven, Belgium. ^49^Department of Pediatrics, Columbia University Irving Medical Center. New York, NY, USA. ^50^University Clinic for Children’s Diseases, Department of Pediatric Immunology, Medical Faculty, University “St.Cyril and Methodij” Skopje, North Macedonia. ^51^Department of Biomedicine, Aarhus University, Aarhus, Denmark; Department of Infectious Diseases, Aarhus University Hospital, Aarhus, Denmark. ^52^Tokyo Medical & Dental University Hospital, Tokyo, Japan. ^53^A*STAR ID labs, Agency for Science, Technology and Research (A*STAR), Singapore; Singapore Immunology Network, Agency for Science, Technology and Research (A*STAR), Singapore. ^54^National Institute of Allergy and Infectious Diseases, National Institutes of Health, Bethesda, MD, USA. ^55^Laboratory of Medical Genetics, IRCCS Bambino Gesù Children’s Hospital, Rome, Italy. ^56^Department of Biomedicine and Prevention, Tor Vergata University of Rome, Rome, Italy. ^57^Department of Pediatrics, Graduate School of Biomedical and Health Sciences, Hiroshima University, Hiroshima, Japan. ^58^Department of Molecular Biology and Genetics, Bilkent University, Bilkent - Ankara, Turkey. ^59^Department of Immunology, University Children’s Hospital Zurich, Zurich, Switzerland. ^60^Department of Biosciences and Nutrition, Karolinska Institutet, Stockholm, Sweden. ^61^Laboratory of Immunogenetics of Human Diseases, Innate Immunity Group, IdiPAZ Institute for Health Research, La Paz Hospital, Madrid, Spain. ^62^IIBB-CSIC, IDIBAPS, Barcelona, Spain. ^63^Faculdades Pequeno Príncipe, Instituto de Pesquisa Pelé Pequeno Príncipe, Curitiba, Brazil. ^64^Neurometabolic Diseases Laboratory, Bellvitge Biomedical Research Institute (IDIBELL), L’Hospitalet de Llobregat, Barcelona, Spain; Catalan Institution of Research and Advanced Studies (ICREA), Barcelona, Spain; Center for Biomedical Research on Rare Diseases (CIBERER), ISCIII, Barcelona, Spain. ^65^Human Evolutionary Genetics Unit, CNRS UMR2000, Institut Pasteur, Paris, France; Human Genomics and Evolution, Collège de France, Paris, France. ^66^Department of Immunology, Hospital Universitario de Gran Canaria Dr. Negrín, Canarian Health System, Las Palmas de Gran Canaria, Spain; Department of Clinical Sciences, University Fernando Pessoa Canarias, Las Palmas de Gran Canaria, Spain. ^67^Imperial College London, London, UK. ^68^Boston Children’s Hospital, Harvard Medical School, Boston, MA, USA. ^69^Saeed Pathobiology and Genetics Lab, Tehran, Iran; Department of Microbiology and Immunology, Clinical and Diagnostic Immunology, KU Leuven, Leuven, Belgium. ^70^Pediatric Infectious Diseases and Immunodeficiencies Unit. Vall d’Hebron Barcelona Hospital Campus. Barcelona, Spain. ^71^St. Giles Laboratory of Human Genetics of Infectious Diseases, Rockefeller Branch, The Rockefeller University, New York, NY, USA; University Medical Center Utrecht, Amsterdam, Netherlands. ^72^Garvan Institute of Medical Research, Darlinghurst, NSW, Australia; St. Vincent’s Clinical School, Faculty of Medicine, UNSW Sydney, NSW, Australia. ^73^Department of Pediatrics, British Columbia Children’s Hospital, The University of British Columbia, Vancouver, BC, Canada. ^74^Holy Family Red Crescent Medical College; Centre for Precision Therapeutics, NeuroGen Children’s Healthcare; Genetics and Genomic Medicine Centre, NeuroGen Children’s Healthcare, Dhaka, Bangladesh. ^75^Mohammed Bin Rashid University of Medicine and Health Sciences, College of Medicine, Dubai, United Arab Emirates; The Centre for Applied Genomics, Department of Genetics and Genome Biology, The Hospital for Sick Children, Toronto, Ontario, Canada. ^76^Amsterdam UMC, University of Amsterdam, Department of Neurology, Amsterdam Neuroscience, Amsterdam, Netherlands. ^77^Department of Medicine, Division of Infectious Diseases, McGill University Health Centre, Montréal, Québec, Canada; Infectious Disease Susceptibility Program, Research Institute, McGill University Health Centre, Montréal, Québec, Canada. ^78^Department of Pediatric Pneumology, Immunology and Intensive Care, Charité Universitätsmedizin, Berlin University Hospital Center, Berlin, Germany; Labor Berlin GmbH, Department of Immunology, Berlin, Germany; Berlin Institutes of Health (BIH), Berlin-Brandenburg Center for Regenerative Therapies, Berlin, Germany. ^79^Molecular Biophysics Division, Faculty of Physics, A. Mickiewicz University, Poznań, Poland. ^80^The Rockefeller University, Howard Hughes Medical Institute, Necker Hospital, New York, NY, USA.

*Leaders of the COVID Human Genetic Effort.

## Appendix II: French COVID Cohort Study Group

Laurent Abel^1^, Claire Andrejak^2^, François Angoulvant^3^, Delphine Bachelet^4^, Romain Basmaci^5^, Sylvie Behillil^6^, Marine Beluze^7^, Dehbia Benkerrou^8^, Krishna Bhavsar^4^, François Bompart^9^, Lila Bouadma^4^, Maude Bouscambert^10^, Mireille Caralp^11^, Minerva Cervantes-Gonzalez^12^, Anissa Chair^4^, Alexandra Coelho^13^, Camille Couffignal^4^, Sandrine Couffin-Cadiergues^14^, Eric D’Ortenzio^12^, Charlene Da Silveira^4^, Marie-Pierre Debray^4^, Dominique Deplanque^15^, Diane Descamps^16^, Mathilde Desvallées^17^, Alpha Diallo^18^, Alphonsine Diouf^13^, Céline Dorival^8^, François Dubos^19^, Xavier Duval^4^, Philippine Eloy^4^, Vincent VE Enouf^20^, Hélène Esperou^21^, Marina Esposito-Farese^4^, Manuel Etienne^22^, Nadia Ettalhaoui^4^, Nathalie Gault^4^, Alexandre Gaymard^10^, Jade Ghosn^4^, Tristan Gigante^23^, Isabelle Gorenne^4^, Jérémie Guedj^24^, Alexandre Hoctin^13^, Isabelle Hoffmann^4^, Salma Jaafoura^21^, Ouifiya Kafif^4^, Florentia Kaguelidou^25^, Sabina Kali^4^, Antoine Khalil^4^, Coralie Khan^17^, Cédric Laouénan^4^, Samira Laribi^4^, Minh Le^4^, Quentin Le Hingrat^4^, Soizic Le Mestre^18^, Hervé Le Nagard^24^, François-Xavier Lescure^4^, Yves Lévy^26^, Claire Levy-Marchal^27^, Bruno Lina^10^, Guillaume Lingas^24^, Jean Christophe Lucet^4^, Denis Malvy^28^, Marina Mambert^13^, France Mentré^4^, Noémie Mercier^18^, Amina Meziane^8^, Hugo Mouquet^20^, Jimmy Mullaert^4^, Nadège Neant^24^, Marion Noret^29^, Justine Pages^30^, Aurélie Papadopoulos^21^, Christelle Paul^18^, Nathan Peiffer-Smadja^4^, Ventzislava Petrov-Sanchez^18^, Gilles Peytavin^4^, Olivier Picone^31^, Oriane Puéchal^12^, Manuel Rosa-Calatrava^10^, Bénédicte Rossignol^23^, Patrick Rossignol^32^, Carine Roy^4^, Marion Schneider^4^, Caroline Semaille^12^, Nassima Si Mohammed^4^, Lysa Tagherset^4^, Coralie Tardivon^4^, Marie-Capucine Tellier^4^, François Téoulé^8^, Olivier Terrier^10^, Jean-François Timsit^4^, Théo Trioux^4^, Christelle Tual^33^, Sarah Tubiana^4^, Sylvie van der Werf^34^, Noémie Vanel^35^, Aurélie Veislinger^33^, Benoit Visseaux^16^, Aurélie Wiedemann^26^, Yazdan Yazdanpanah^36^

^1^Inserm UMR 1163, Paris, France. ^2^CHU Amiens, France. ^3^Hôpital Necker, Paris, France. ^4^Hôpital Bichat, Paris, France. ^5^Hôpital Louis Mourrier, Colombes, France. ^6^Institut Pasteur, Paris, France. ^7^F-CRIN Partners Platform, AP-HP, Université de Paris, Paris, France. ^8^Inserm UMR 1136, Paris, France. ^9^Drugs for Neglected Diseases Initiative, Geneva, Switzerland. ^10^Inserm UMR 1111, Lyon, France. ^11^Inserm Transfert, Paris, France. ^12^REACTing, Paris, France. ^13^Inserm UMR 1018, Paris, France. ^14^Inserm, Pôle Recherche Clinique, Paris, France. ^15^CIC 1403 Inserm-CHU Lille, Paris, France. ^16^Université de Paris, IAME, INSERM UMR 1137, AP-HP, University Hospital Bichat Claude Bernard, Virology, Paris, France. ^17^Inserm UMR 1219, Bordeaux, France. ^18^ANRS, Paris, France. ^19^CHU Lille, Lille, France. ^20^Pasteur Institute, Paris, France. ^21^Inserm sponsor, Paris, France. ^22^CHU Rouen–SMIT, Rouen, France. ^23^FCRIN INI-CRCT, Nancy, France. ^24^Inserm UMR 1137, Paris, France. ^25^Centre d’Investigation Clinique, Inserm CIC1426, Hôpital Robert Debré, Paris, France. ^26^Inserm UMR 955, Créteil, France; Vaccine Research Instiute (VRI), Paris, France. ^27^F-CRIN INI-CRCT, Paris, France. ^28^CHU de Bordeaux–SMIT, Bordeaux, France. ^29^RENARCI, Annecy, France. ^30^Hôpital Robert Debré, Paris, France. ^31^Hôpital Louis Mourier–Gynécologie, Colombes, France. ^32^University of Lorraine, Plurithematic Clinical Investigation Centre Inserm CIC-P; 1433, Inserm U1116, CHRU Nancy Hopitaux de Brabois, F-CRIN INI-CRCT (Cardiovascular and Renal Clinical Trialists), Nancy, France. ^33^Inserm CIC-1414, Rennes, France. ^34^Institut Pasteur, UMR 3569 CNRS, Université de Paris, Paris, France. ^35^Hôpital la Timone, Marseille, France. ^36^Bichat–SMIT, Paris, France.

## Appendix III: CoV-Contact Cohort

Loubna Alavoine^1^, Karine K. A. Amat^2^, Sylvie Behillil^3^, Julia Bielicki^4^, Patricia Bruijning^5^, Charles Burdet^6^, Eric Caumes^7^, Charlotte Charpentier^8^, Bruno Coignard^9^, Yolande Costa^1^, Sandrine Couffin-Cadiergues^10^, Florence Damond^8^, Aline Dechanet^11^, Christelle Delmas^10^, Diane Descamps^8^, Xavier Duval^1^, Jean-Luc Ecobichon^1^, Vincent Enouf^3^, Hélène Espérou^10^, Wahiba Frezouls^1^, Nadhira Houhou^11^, Emila Ilic-Habensus^1^, Ouifiya Kafif^11^, John Kikoine^11^, Quentin Le Hingrat^8^, David Lebeaux^12^, Anne Leclercq^1^, Jonathan Lehacaut^1^, Sophie Letrou^1^, Bruno Lina^13^, Jean-Christophe Lucet^14^, Denis Malvy^15^, Pauline Manchon^11^, Milica Mandic^1^, Mohamed Meghadecha^16^, Justina Motiejunaite^17^, Mariama Nouroudine^1^, Valentine Piquard^11^, Andreea Postolache^11^, Caroline Quintin^1^, Jade Rexach^1^, Layidé Roufai^10^, Zaven Terzian^11^, Michael Thy^18^, Sarah Tubiana^1^, Sylvie van der Werf^3^, Valérie Vignali^1^, Benoit Visseaux^8^, Yazdan Yazdanpanah^14^

^1^Centre d’Investigation Clinique, Inserm CIC 1425, Hôpital Bichat Claude Bernard, APHP, Paris, France. ^2^IMEA Fondation Léon M’Ba, Paris, France. ^3^Institut Pasteur, UMR 3569 CNRS, Université de Paris, Paris, France. ^4^University of Basel Children’s Hospital. ^5^Julius Center for Health Sciences and Primary Care, Utrecht, Netherlands. ^6^Université de Paris, IAME, Inserm UMR 1137, F-75018, Paris, France; Hôpital Bichat Claude Bernard, APHP, Paris, France. ^7^Hôpital Pitiè Salpétriere, APHP, Paris. ^8^Université de Paris, IAME, INSERM UMR 1137, AP-HP, University Hospital Bichat Claude Bernard, Virology, Paris, France. ^9^Santé Publique France, Saint Maurice, France. ^10^Pole Recherche Clinique, Inserm, Paris, France. ^11^Hôpital Bichat Claude Bernard, APHP, Paris, France. ^12^APHP, Paris, France. ^13^Virpath Laboratory, International Center of Research in Infectiology, Lyon University, INSERM U1111, CNRS UMR 5308, ENS, UCBL, Lyon, France. ^14^IAME Inserm UMR 1138, Hôpital Bichat Claude Bernard, APHP, Paris, France. ^15^Service des Maladies Infectieuses et Tropicales; Groupe Pellegrin-Place Amélie-Raba-Léon, Bordeaux, France. ^16^Hôpital Hotel Dieu, APHP, Paris, France. ^17^Service des Explorations Fonctionnelles, Hôpital Bichat–Claude Bernard, APHP, Paris, France. ^18^Center for Clinical Investigation, Assistance Publique-Hôpitaux de Paris, Bichat-Claude Bernard University Hospital, Paris, France.
