# Supplementary material for: Inhibition of HECT E3 ligases as potential therapy for COVID-19
Source: Cell Death Dis. 2021 Mar 24;12(4):310. doi: 10.1038/s41419-021-03513-1 (PMC7987752; doi:10.1038/s41419-021-03513-1)
Supplement: Supplementary file 2 — Supplementary Table 2 [file 41419_2021_3513_MOESM2_ESM.pdf]

Gene	Variant	Gnomadv2.1 frequency	CADD	Mutation significance cut-off 95% (Itan et al, Nature methods 2016)
HECW1	p.Asp828Glu	0	24,1	11,67
HECW2	p.Pro134Ser	0,001766609	22,1	27,11
HECW2	p.Thr116Ile	0,004761433	26,7	27,11
HECW2	p.Val573Ile	8,58882E-05	14,98	27,11
NEDD4	p.Asp129Asn	0,000294487	14,76	16,74
NEDD4	p.Gly451Ala	0,002257568	6,025	16,74
NEDD4	p.Leu687Phe	0,000627339	23,2	16,74
NEDD4	p.Pro539del	0,001141074	21,5	16,74
NEDD4	p.Ile843Arg	3,53689E-05	23,4	16,74
NEDD4	p.Arg877Gln	9,22483E-05	33	16,74
SMURF2	p.Gly10Glu	0,003192598	13,29	28,65
WWP1	p.Asn745Ser	0,001614022	18,89	15,45
WWP2	p.Lys620Asn	0,000127496	26,6	13,9
