# Supplementary material for: Inhibition of HECT E3 ligases as potential therapy for COVID-19
Source: Cell Death Dis. 2021 Mar 24;12(4):310. doi: 10.1038/s41419-021-03513-1 (PMC7987752; doi:10.1038/s41419-021-03513-1)
Supplement: Supplementary file 3 — Supplementary Table 3 [file 41419_2021_3513_MOESM3_ESM.pdf]

Gene	SNP	Gnomad AF	most_severe_consequence	Sift prediction	Polyphen	HGVSP	Gender	Age	Outcome
HECW1	chr7:43550491	<0.01	missense_variant	deleterious	possibly_damaging	T1432M	M	77	Survived
HECW1	chr7:43456393	<0.01	missense_variant	tolerated	benign	P866L	F	48	Survived
NEDD4	chr15:55916758	<0.01	missense_variant	deleterious_low_confidence	possibly_damaging	D25G	M	74	Survived
NEDD4	chr15:55833109	<0.01	splice_region_variant			c.3688-5T>C	N.A.	N.A.	N.A.
NEDD4	chr15:55951530	<0.01	missense_variant	tolerated	benign	Q60R	F	77	Survived
NEDD4L	chr18:58333891	0	missense_variant	tolerated	benign	P355L	M	63	Survived
NEDD4L	chr18:58349550	0	missense_variant	tolerated	benign	L530S	F	62	Survived
WWP2	chr16:69842059	<0.01	missense_variant	tolerated	benign	A172P	F	72	Survived
WWP2	chr16:69842128	<0.01	splice_region_variant			c.575+8C>G	M	24	Survived
