# Supplementary figures and images for: Inhibition of HECT E3 ligases as potential therapy for COVID-19
Source: Cell Death Dis. 2021 Mar 24;12(4):310. doi: 10.1038/s41419-021-03513-1 (PMC7987752; doi:10.1038/s41419-021-03513-1)

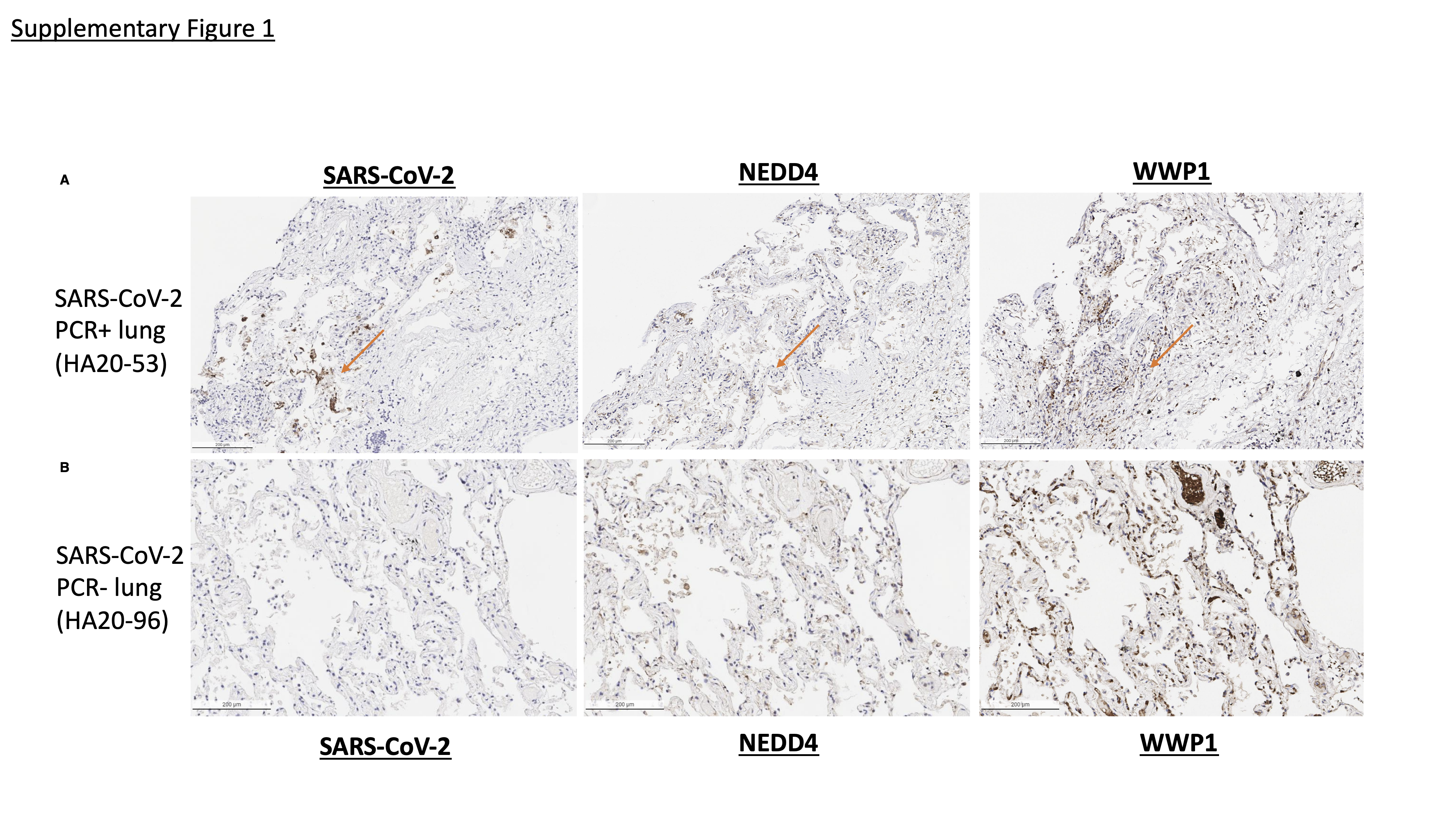

Supplement: Supplementary file 4 — Supplementary Figure 1 [file 41419_2021_3513_MOESM4_ESM.png]

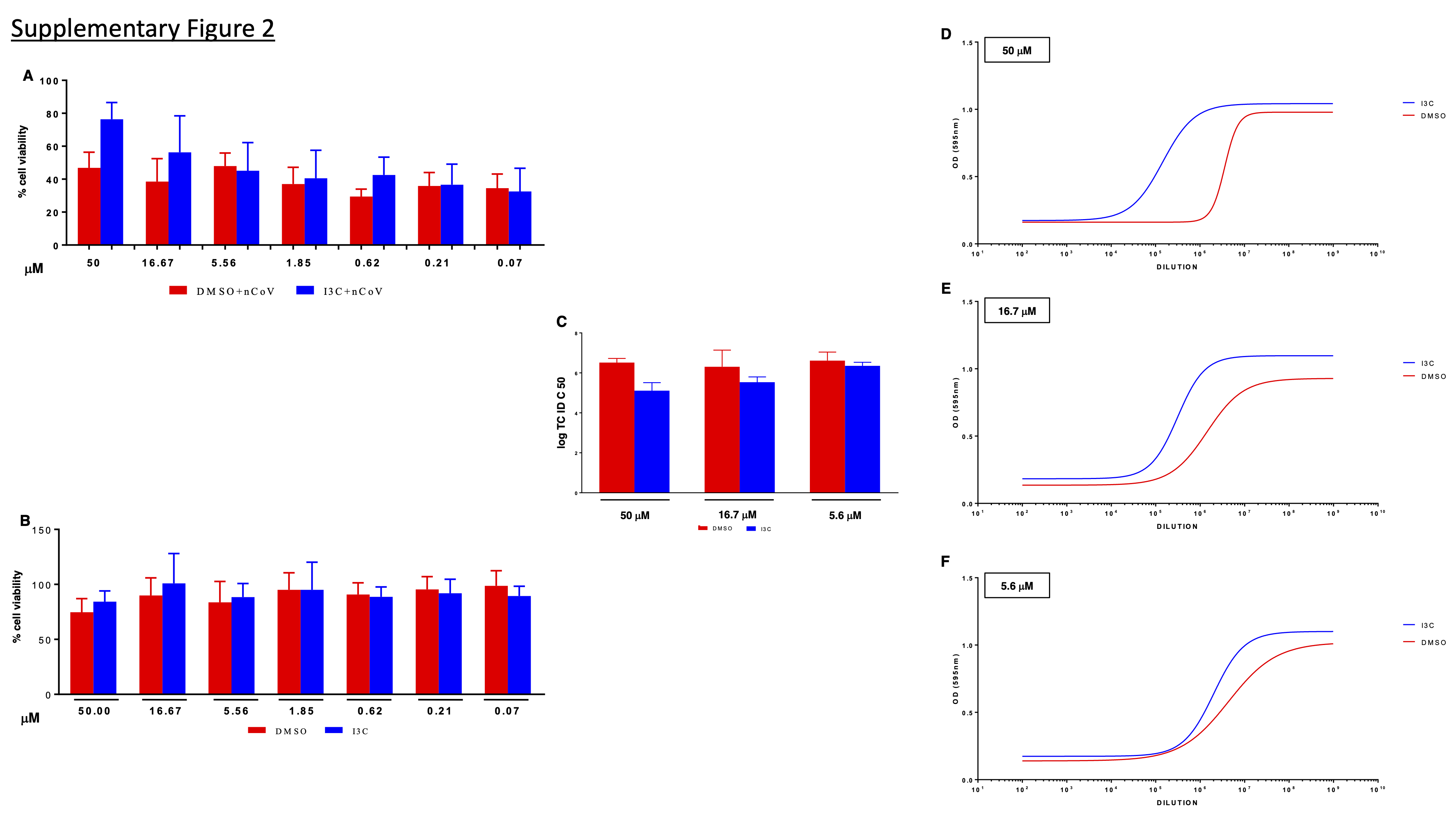

Supplement: Supplementary file 5 — Supplementary Figure 2 [file 41419_2021_3513_MOESM5_ESM.png]

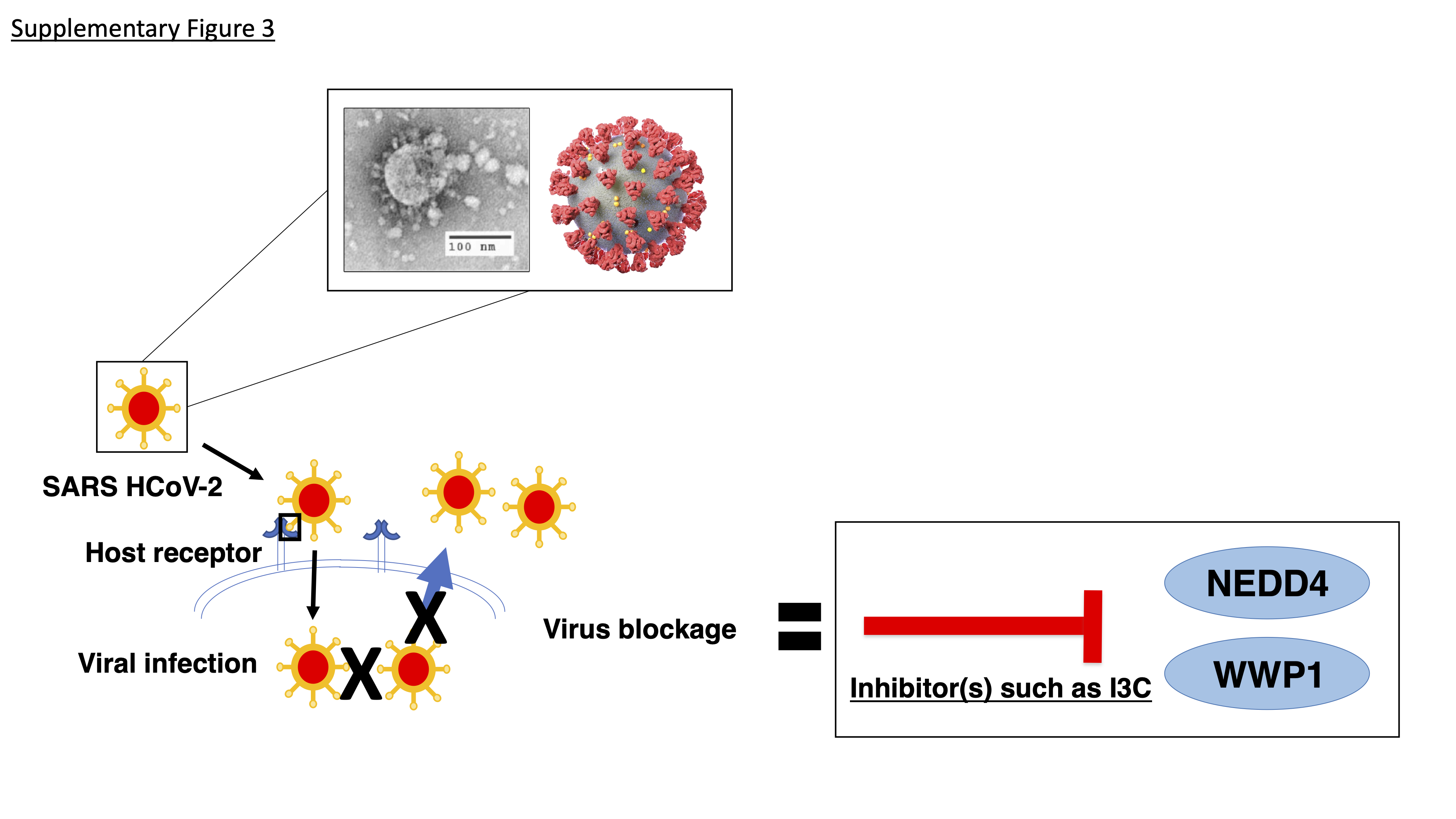

Supplement: Supplementary file 6 — Supplementary Figure 3 [file 41419_2021_3513_MOESM6_ESM.png]
