# Supplementary material for: Inhibition of HECT E3 ligases as potential therapy for COVID-19
Source: Cell Death Dis. 2021 Mar 24;12(4):310. doi: 10.1038/s41419-021-03513-1 (PMC7987752; doi:10.1038/s41419-021-03513-1)
Supplement: Supplementary file 7 — Related Manuscript File [file 41419_2021_3513_MOESM7_ESM.pdf]

DECLARATION OF CONTRIBUTIONS TO ARTICLE

ADMC

Manuscript Number: CDDIS-21-0402R

Journal Name: *Cell Death & Disease* (the 'Journal')

Proposed Title of the Contribution:

Inhibition of HECT E3 ligases as potential therapy for COVID-19 (the 'Contribution)

Author(s):

Giuseppe Novelli, Jing Liu, Michela Biancolella, Tonino Alonzi, Antonio Novelli, J.J. Patten, Dario (the 'Authors')

For all *CDDis* articles, each person named as an author in the published version must be able to show he or she has contributed substantially to the article.

Authorship credit should be based on 1) substantial contributions to conception and design, acquisition of data, or analysis and interpretation of data; 2) drafting the article or revising it critically for important intellectual content; and 3) final approval of the version to be published. Authors should meet conditions 1, 2 and 3.

Any person who cannot be shown to have made a substantial contribution to the article cannot be listed as an author in the final version. The name of any person who is deemed to have made a minor contribution can, however, appear in the Acknowledgments section of the article.

Please complete the table below to indicate the contributions of all named authors to the manuscript.

Author Full Name:	Specification of Contribution to the Manuscript:
G. Novelli; P. P. Pandolfi	Wrote the draft of the paper in particular genetics section, and
J. Liu; W. Wei	Description of the functional studies using HECT proteins
J- L.Casanova; L. Abel; A.Cobat	Wrote the section of the analysis and interpretation of genetic data
J.J.Grzymski; G.Elhanan	Genetic Analysis and interpretation of genetic data
M. Biancolella; B. Rizzacasa; S. Grelli	Performed RNA expression analysis and statistical tests and decribed the specific section
T. Alonzi; M.R. Capobianchi;D.Goletti;J.J.Patten; R.A. Davey	Provided in vivo SARS-CoV-2 assays and inhibition tests
K.Cheng; J.Mann; T. D. McKee; F. Krammer; F.Amanat	Performed histological analysis in human lung tissue and in
G. Pepe; A. Guarracino	In silico analysis of structural proteins
H.C. Su; L.D. Notarangelo	Genetic data Analysis; Review of the draft
Y.T.Lambiotte; Y. Uzunhan; S. Tubiana; J.Ghosn	Provided clinical data
A. Novelli; D.Cocciadiferro; E. Agolini; B. Bigio; V.L.Colona; R.Giannini; A. Latini	WES Analysis, genetic study and clinical correlation
C. Tomino; S. Jain; S.Sidhu	I3C chemical study, biochemical analysis

Please complete the table below to indicate the contributions of all named authors to the figures.

Figure 1:

Wenyi Wei; Jing Liu

Figure 2:

Michela Biancolella; Barbara Rizzacasa; Sandro Grelli

Figure 3:

Justin Mann, Trevor D. McKee, Ke Cheng

Figure 4:

Andrea Guarracino, Gerardo Pepe

Figure 5:

Wenyi Wei; Jing Liu; Pier Paolo Pandolfi

Figure 6:

Delia Goletti, Maria Rosaria Capobianchi; Tonino Alonzi

Signed for and on behalf of the Author(s):	Print Name:	Date:
Guell	Giuseppe Novelli	February, 5, 2021
