# Supplementary material for: Inhibition of HECT E3 ligases as potential therapy for COVID-19
Source: Cell Death Dis. 2021 Mar 24;12(4):310. doi: 10.1038/s41419-021-03513-1 (PMC7987752; doi:10.1038/s41419-021-03513-1)
Supplement: Supplementary file 9 — Supplementary Figure legends [file 41419_2021_3513_MOESM9_ESM.docx]

**Supplementary Figure 1.** A-B) Regions of human autopsy lungs expressing higher SARS-CoV-2 nucleocapsid protein (upper low) show higher NEDD4 and WWP1 in consecutive sections (10X mag, n.s.). Autopsy lungs from SARS-CoV-2 PCR negative cases show high basal levels of NEDD4 and WWP1.

**Supplementary Figure 2.** I3C inhibited SARS-CoV-2-induced CPE and viral production in Vero E6 cells infected at a 10-fold higher MOI. Cells were treated with different doses of I3C (from 50 to 0.069 μM; 1:3 serial dilutions) or DMSO (from 0.5 to 6.9x10-4 v/v percentage) 1h before SARS-CoV-2 infection (MOI=0.01) in triplicates. Absorption of the virus was allowed for 1h at 37oC in presence of I3C or DMSO treatments. The unabsorbed virus was removed and replaced by fresh medium with I3C or DMSO as above. Cells were then treated with either I3C or DMSO after 24h and incubated at 37°C with 5% CO2 for 48h when the survival of infected (A) or not infected (B) cells was measured by crystal violet staining assay. The results were evaluated setting the not infected cells as 100% and the remaining values represented as a relative value. Experiments were performed in triplicate and data are expressed as mean S.D. (n = 2). Culture media of SARS-CoV-2-infected cells, treated as above, were collected and progeny titres were assessed with the CPE inhibition assay using Vero E6 cells. Survival of the cells was measured by crystal violet staining assay. Results were analysed using Graph Pad (GraphPad Prism 8 XML ProjecT) with nonlinear regression curve fit (Inhibitor vs. response-Variable slope (four parameters)) ((D-F) and data presented as logIC50 (C).

**Supplementary Figure 3**. HECT Type E3 Ubiquitin Ligases: critical and druggable therapeutic targets for viral egression and replication.
